# Non-Necroptotic Roles of MLKL in Diet-Induced Obesity, Liver Pathology, and Insulin Sensitivity: Insights from a High-Fat, High-Fructose, High-Cholesterol Diet Mouse Model

**DOI:** 10.3390/ijms25052813

**Published:** 2024-02-28

**Authors:** Phoebe Ohene-Marfo, Hoang Van M. Nguyen, Sabira Mohammed, Nidheesh Thadathil, Albert Tran, Evan H. Nicklas, Dawei Wang, Ramasamy Selvarani, Jacob W. Farriester, Rohan Varshney, Michael Kinter, Arlan Richardson, Michael C. Rudolph, Sathyaseelan S. Deepa

**Affiliations:** 1 Department of Biochemistry and Physiology, University of Oklahoma Health Sciences Center, Oklahoma City, OK 73104, USA; phoebe-ohenemarfo@ouhsc.edu (P.O.-M.); nidheesh-thadathil@ouhsc.edu (N.T.); albert-tran@ouhsc.edu (A.T.); enicklas7@gmail.com (E.H.N.); davidwangoita@gmail.com (D.W.); ramasamy-selvarani@ouhsc.edu (R.S.); jacob-farriester@ouhsc.edu (J.W.F.); rohan-varshney@ouhsc.edu (R.V.); arlan-richardson@ouhsc.edu (A.R.); michael-rudolph@ouhsc.edu (M.C.R.); 2 Department of Nutritional Sciences, University of Oklahoma Health Sciences Center, Oklahoma City, OK 73104, USA; hoangvan-nguyen@ouhsc.edu; 3 Stephenson Cancer Center, University of Oklahoma Health Sciences Center, Oklahoma City, OK 73104, USA; sabira-jazir@ouhsc.edu; 4 Harold Hamm Diabetes Center, University of Oklahoma Health Sciences Center, Oklahoma City, OK 73104, USA; 5 Aging and Metabolism Research Program, Oklahoma Medical Research Foundation, Oklahoma City, OK 73104, USA; mike-kinter@omrf.org; 6 Oklahoma Center for Geroscience & Brain Aging, University of Oklahoma Health Sciences Center, Oklahoma City, OK 73104, USA; 7 Oklahoma City VA Medical Center, Oklahoma City, OK 73104, USA

**Keywords:** insulin sensitivity, liver fibrosis, MAFLD, MLKL, obesity

## Abstract

Chronic inflammation is a key player in metabolic dysfunction-associated fatty liver disease (MAFLD) progression. Necroptosis, an inflammatory cell death pathway, is elevated in MAFLD patients and mouse models, yet its role is unclear due to the diverse mouse models and inhibition strategies. In our study, we inhibited necroptosis by targeting mixed lineage kinase domain-like pseudokinase (MLKL), the terminal effector of necroptosis, in a high-fat, high-fructose, high-cholesterol (HFHFrHC) mouse model of diet-induced MAFLD. Despite the HFHFrHC diet upregulating MLKL (2.5-fold), WT mice livers showed no increase in necroptosis markers or associated proinflammatory cytokines. Surprisingly, *Mlkl^−/−^* mice experienced exacerbated liver inflammation without protection from diet-induced liver damage, steatosis, or fibrosis. In contrast, *Mlkl^+/−^* mice showed a significant reduction in these parameters that was associated with elevated Pparα and Pparγ levels. Both *Mlkl^−/−^* and *Mlkl^+/−^* mice on the HFHFrHC diet resisted diet-induced obesity, attributed to the increased beiging, enhanced oxygen consumption, and energy expenditure due to adipose tissue, and exhibited improved insulin sensitivity. These findings highlight the tissue-specific effects of MLKL on the liver and adipose tissue, and they suggest a dose-dependent effect of MLKL on liver pathology.

## 1. Introduction

Metabolic-associated fatty liver disease (MAFLD) is a prevalent global liver condition linked to obesity, type 2 diabetes, and insulin resistance [1]. MAFLD covers a spectrum of liver diseases, ranging from fat deposition in the liver (steatosis) to metabolic dysfunction-associated steatohepatitis (MASH), which is characterized by fat accumulation (steatosis) in >5% of hepatocytes, and inflammation with hepatocyte injury (ballooning) with varying degrees of fibrosis [2]. The obesity epidemic has led to a rapid increase in MAFLD’s prevalence, affecting nearly 38% of the population [3]. Around 30% of those with steatosis progress to MASH, a significant cause of liver-related morbidity and mortality. MASH is a leading factor in liver transplantation cases and a major risk factor for hepatocellular carcinoma (HCC) and cardiovascular diseases [4,5]. Despite ongoing clinical trials, there is currently no approved pharmacological approach against MASH, emphasizing the need for a deeper understanding of its pathogenic mechanisms.

One of the factors that differentiates MASH from simple fatty liver is the occurrence of massive hepatocyte cell death in MASH and the subsequent increase in liver inflammation and fibrosis [6]. Hepatocyte cell death is a critical event in the development and progression of MAFLD because it triggers inflammation leading to fibrosis. Apoptosis is a major form of cell death in liver diseases; however, in recent years, necroptosis has emerged as an important form of programmed cell death in the liver [7]. Apoptotic cell death is characterized by lower levels of inflammation, whereas necroptosis induces significant inflammation due to the release of damage-associated molecular patterns (DAMPs). Necroptosis is initiated when necroptotic stimuli (e.g., oxidative stress, TNFα, mTOR activation, lipotoxicity, etc.) sequentially activate receptor-interacting serine/threonine kinase 1 (RIPK1), RIPK3, and mixed lineage kinase domain-like pseudokinase (MLKL) through phosphorylation. Phosphorylated MLKL undergoes oligomerization, leading to membrane permeabilization and release of DAMPs, which bind to the cell surface receptors on innate immune cells, promoting increased transcription of proinflammatory cytokines causing inflammation [8,9]. Studies have shown that markers of necroptosis (RIPK3, MLKL, and phospho-MLKL) are increased in the livers of MAFLD and MASH patients, and in mouse models of MASH [10,11,12,13,14,15]. Consequently, inhibiting necroptosis, either through pharmacological targeting of RIPK1 or genetic/pharmacological targeting of RIPK3, has been demonstrated to decrease liver inflammation and fibrosis in mouse models of MAFLD [12,16,17]. We have previously shown that the inhibition of necroptosis using necrostatin-1s, a pharmacological inhibitor of RIPK1, reduced liver inflammation and fibrosis in mouse models of spontaneous MASH, i.e., old wild-type mice and *Sod1^−/−^* mice [18,19]. Thus, these studies provide evidence for the involvement of necroptosis-mediated inflammation in the pathology of MAFLD/MASH. However, conflicting findings are present in the literature concerning the effects of *Ripk3* or *Mlkl* deletion on liver inflammation and/or fibrosis, with outcomes differing based on the dietary model of MAFLD.

In a methionine choline-deficient (MCD) or choline-deficient high-fat diet (CD-HFD, 35% fat) non-obese model, RIPK3 deletion reduces steatosis, inflammation, and fibrosis [12,14]. Conversely, in an HFD-induced obesity-driven MAFLD, RIPK3 deletion exacerbates outcomes, including increased body weight, insulin resistance, glucose intolerance, liver steatosis, inflammation, and fibrosis [13,20]. Thus, RIPK3′s role in liver inflammation and fibrosis is model-dependent and influenced by diet composition. Similarly, MLKL loss also yields varied outcomes in MAFLD mouse models: *Mlkl* deletion reduces liver inflammation in HFD or Western diet-induced MAFLD mouse models [15,21]; however, Xu et al., (2018) reported no impact of *Mlkl* deletion on liver inflammation in an HFD-induced model of MAFLD [22]. Notably, the effect of targeting *Mlkl* on liver fibrosis in diet-induced MAFLD mouse models is unclear. Recently, we have shown that in a CD-HFD (60% fat) non-obese model, either *Ripk3* or *Mlkl* deletion reduced liver inflammation and HCC, whereas liver fibrosis was not reduced [23]. Thus, the existence of a knowledge gap regarding the role of necroptosis in obesity-driven MASH is currently unresolved.

The primary goal of this study is to investigate the role of MLKL, the key effector molecule in the necroptosis pathway, in obesity-driven MASH. Specifically, the study aims to elucidate how MLKL deficiency, achieved through the use of *Mlkl^−/−^* and *Mlkl^+/−^* mice, impacts liver injury, fibrosis, and metabolic outcomes associated with MAFLD. By addressing this question, this study seeks to contribute to a better understanding of the mechanisms underlying liver pathology in the context of obesity-driven MASH, potentially uncovering new therapeutic targets for intervention. To address this, we investigated the impact of the absence of *Mlkl* (*Mlkl^−/−^* mice) or partial loss of *Mlkl* (*Mlkl^+/−^* mice) on MAFLD using a diet that mediates obesity-driven MAFLD, viz., a high-fat, high-fructose, high-cholesterol (HFHFrHC) diet that is documented to promote obesity-mediated MAFLD [24]. Our data show that the absence of MLKL did not protect against liver injury or fibrosis induced by the HFHFrHC diet. However, a partial loss of *Mlkl* protected from HFHFrHC diet-induced liver injury and fibrosis. Interestingly, both *Mlkl^−/−^* and *Mlkl^+/−^* mice displayed resistance to HFHFrHC diet-induced obesity and improved insulin sensitivity compared to control wild-type mice (WT, *Mlkl^+/+^*) fed the same diet.

## 2. Results

### 2.1. Mlkl^−/−^ and Mlkl^+/−^ Mice Are Resistant to Diet Induced Obesity

**Figure 1:**
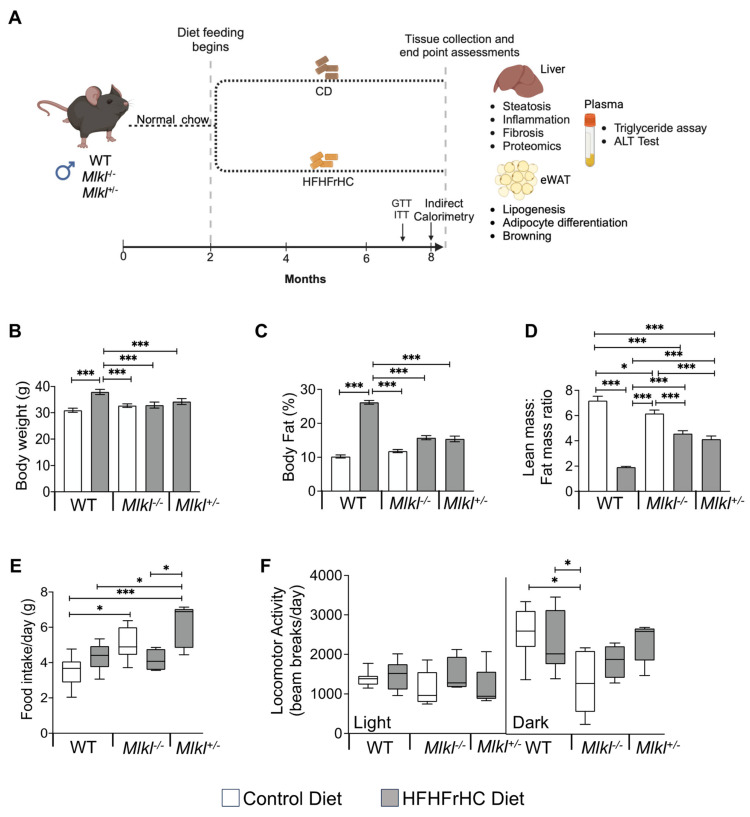
*Mlkl^−/−^* and *Mlkl^+/−^* mice are resistant to diet-induced obesity. (**A**) Schematic representation of the experimental design. Male mice were fed CD (WT, *Mlkl^−/−^* or *Mlkl^+/−^*) or HFHFrHC diet (WT, *Mlkl^−/−^* or *Mlkl^+/−^*) from 2 months of age for 6 months. Metabolic assessment studies (GTT, ITT, indirect calorimetry) and body composition analysis were performed at the 5th and 6th month of diet-feeding, respectively. At 8 months of age (6 months after diet-feeding), liver, white adipose tissue, and plasma were collected from each mouse for biochemical analysis (*n* = 7–8 for WT; 5–6 for *Mlkl^−/−^* or *Mlkl^+/−^*). (**B**) Body weight of mice represented in grams; percentage of fat mass normalized to total body weight (**C**) and ratio of lean mass to fat mass in grams (**D**). (**E**) Average daily food intake during and light and dark cycles combined. (**F**) Average daily spontaneous locomotor activity during light and dark phases of the light cycle. White bars/box plots represent CD and gray bars/box plots represent HFHFrHC diet. Error bars are represented as mean ± SEM. One-way ANOVA, * *p* < 0.05, *** *p* < 0.0005.

The experimental design is shown in Figure 1A, where WT, *Mlkl^−/−^* and *Mlkl^+/−^* male mice were fed the HFHFrHC diet for 6 months, starting at 2 months of age. Feeding HFHFrHC diet resulted in a significant increase (15.4%) in total body weight. Notably, this weight gain was not observed in *Mlkl^−/−^* or *Mlkl^+/−^* mice (Figure 1B and Figure S1A). Compared to Control Diet (CD, open bars)-fed WT mice, HFHFrHC diet (grey bars)-fed WT mice had a 2.6-fold increase in the percentage fat mass. However, either the absence or partial loss of *Mlkl* (*Mlkl^−/−^* or *Mlkl^+/−^*) led to significant attenuation of fat deposition (Figure 1C). The lean mass to fat mass ratio was significantly lower in WT mice fed the HFHFrHC diet, whereas this effect was attenuated in *Mlkl^−/−^* or *Mlkl^+/−^* mice (Figure 1D). No significant differences in food or water intake were observed between WT or *Mlkl^−/−^* mice provided either CD or HFHFrHC diet (Figure 1E and Figure S1B–D). CD-fed *Mlkl^−/−^* mice exhibited a significant reduction in ambulatory activity during the dark phase compared to both CD-fed or HFHFrHC diet-fed WT groups (Figure 1F and Figure S1E). Together, based on the increased body weight and adipose accumulation, diet-induced obesity in HFHFrHC diet-fed WT mice is attenuated in *Mlkl^−/−^* and *Mlkl^+/−^* mice and is not accounted for by changes in energy intake or ambulatory activity.

### 2.2. Mlkl^+/−^ Mice, Not Mlkl^−/−^ Mice, Are Protected from Diet-Induced Hepatic Injury and Steatosis

**Figure 2:**
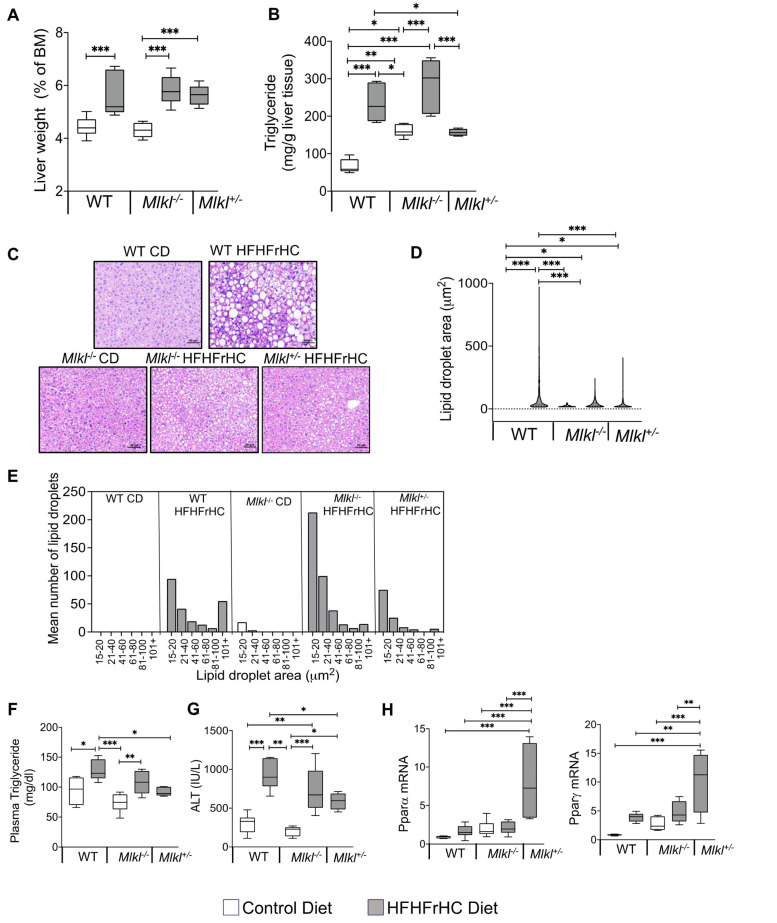
*Mlkl^+/−^* mice, not *Mlkl^−/−^* mice, are protected from diet-induced hepatic injury and steatosis. Data from WT, *Mlkl^−/−^* or *Mlkl*^+/−^ mice fed a CD (white bars/box plots) or HFHFrHC diet (gray bars/box plots). (**A**) Percentage of liver weight normalized to body mass (BM). (**B**) Quantification of total triglyceride in liver tissue (mg/g). (**C**) Representative images of H&E-stained liver sections. Scale bar: 50 μM (*n* = 3 per group). (**D**) Mean lipid droplet area in the livers of WT, *Mlkl^−/−^* or *Mlkl^+/−^* (*n* = 3 per group). (**E**) Frequency distribution of lipid droplets based on their size in the livers of WT, *Mlkl^−/−^* or *Mlkl^+/−^* (*n* = 3 per group); quantification of total triglyceride in plasma (mg/dL) (**F**). (**G**) Levels of ALT (IU/L) in plasma. (**H**) The mRNA levels of Pparα and Pparγ represented as the fold change normalized to β-microglobulin (*n* = 7–8 for WT; 5–8 for *Mlkl^−/−^* or *Mlkl^+/−^*). Error bars are represented as mean ±SEM. One-way ANOVA *p* < 0.05. One-way ANOVA *p* < 0.05. * *p* < 0.05, ** *p* < 0.005, *** *p* < 0.0005.

The HFHFrHC diet-feeding resulted in a significant increase in the percentage liver weights of WT, *Mlkl^−/−^* and *Mlkl^+/−^* mice (~1.3-fold) (Figure 2A). As anticipated, consumption of the HFHFrHC diet resulted in a significant increase in the MLKL protein (2.5-fold) expression in WT mice. However, unexpectedly, a 50% reduction in RIPK3 protein and no change in phospho-MLKL and MLKL oligomers (markers of necroptosis) were observed (Figure S2A,B). Feeding the HFHFrHC diet increased the levels of triglycerides in the liver (2–2.5-fold) (Figure 2B), and H&E staining revealed the accumulation of enlarged lipid droplets in the liver of WT mice fed the HFHFrHC diet compared to WT mice fed CD, indicating increased steatosis (Figure 2C,D). *Mlkl^−/−^* mice fed the HFHFrHC diet had smaller-sized lipid droplets in the liver; however, the number of lipid droplets was ~2-fold greater than found in either the WT or *Mlkl^+/−^* HFHFrHC diet groups (Figure 2E). In contrast, *Mlkl^+/−^* mice on the HFHFrHC diet had fewer and smaller-sized lipid droplets relative to the WT HFHFrHC group (Figure 2B–E). The plasma triglyceride levels increased 1.5-fold, as did the plasma ALT levels (2-fold), in the WT HFHFrHC group (Figure 2F,G). The absence of MLKL (*Mlkl^−/−^* mice) did not reduce the plasma triglyceride or ALT levels; however, the partial loss of MLKL (*Mlkl^+/−^* mice) had significantly lower plasma triglycerides and ALT (Figure 2F,G). In line with this observation, the transcript levels of Pparα and Pparγ, two key regulators of lipid metabolism [25,26], were significantly elevated in the livers of *Mlkl^+/−^* mice compared to the other experimental groups (Figure 2H).

**Figure 3:**
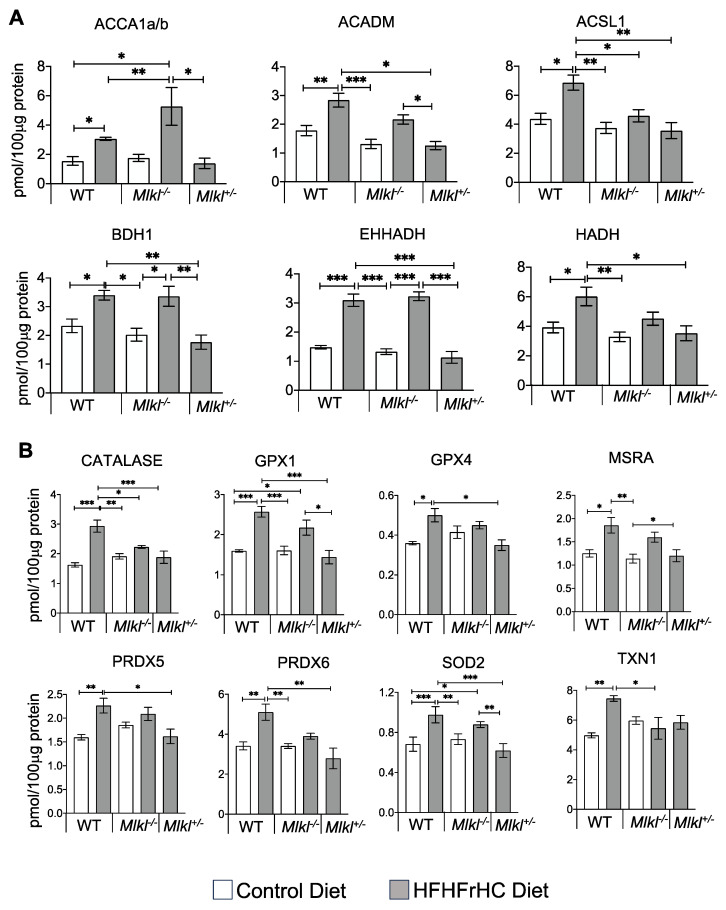
Liver proteomics data. Graphical representation of enzymes involved in fatty acid oxidation (**A**) and proteins associated with oxidative stress (**B**) in the livers of WT, *Mlkl^−/−^* or *Mlkl^+/−^* mice fed a CD (white bar) or HFHFrHC diet (gray bar) (*n* = 5/group) measured by mass spectrometry analysis. Error bars are represented as mean ±SEM. One-way ANOVA *p* < 0.05, * *p* < 0.05, ** *p* < 0.005, *** *p* < 0.0005. ACCA1a/b: acetyl-CoA carboxylase alpha and beta, ACADM: medium-chain acyl-CoA dehydrogenase, ACSL1: acyl-CoA synthetase long-chain family member 1, BDH1: 3-hydroxybutyrate dehydrogenase 1, EHHADH: enoyl-CoA hydratase and 3-hydroxyacyl CoA dehydrogenase, HADH: 3-hydroxyacyl-CoA dehydrogenase, GPX: glutathione peroxidase, MSRA: methionine sulfoxide reductase A, PRDX: peroxiredoxin, SOD2: superoxide dismutase 2, TXN1: thioredoxin 1.

Next, we conducted targeted proteomic analysis to assess the effect of the absence or reduction of *Mlkl* on the proteins involved in mitochondrial fatty acid oxidation (Figure 3A). The WT HFHFrHC diet group had significant increases in the protein levels of key fatty acid oxidation pathway enzymes, including acetyl-CoA carboxylase alpha and beta (ACCA1a/b, facilitating fatty acid oxidation), acyl-CoA synthetase long-chain family member 1 (ACSL1, involved in activating long-chain fatty acids for beta-oxidation), medium-chain acyl-CoA dehydrogenase (ACADM, participating in fatty acid beta-oxidation), enoyl-CoA hydratase and 3-hydroxyacyl CoA dehydrogenase (EHHADH, contributing to fatty acid beta-oxidation), 3-hydroxybutyrate dehydrogenase 1 (BDH1, involved in ketone body metabolism), and 3-hydroxyacyl-CoA dehydrogenase (HADH, engaged in fatty acid beta-oxidation). While ACC1a/b, BDH1, and EHHADH were also significantly increased in the *Mlkl^−/−^* group during the HFHFrHC feeding, the abundance of BDH1 and EHHADH was decreased significantly in the *Mlkl^+/−^* HFHFrHC group relative to the WT HFHFrHC group (except for ACCA1a/b). Cumulatively, the absence or partial loss of MLKL had distinct effects on proteins regulating fatty acid metabolism compared to mice with intact MLKL.

Fatty acid oxidation is known to elevate mitochondrial reactive oxygen species (ROS) levels, inducing oxidative stress [27], which is a key contributor to MAFLD development [28]. Therefore, we evaluated antioxidant enzyme levels, as tissues upregulate these enzymes to protect against ROS and serve as a useful predictor of oxidative stress [29,30]. Proteomic analysis revealed significant increase in the key antioxidant enzymes in response to the HFHFrHC diet-feeding in WT mice, including superoxide dismutase 2 (SOD2, 1.6-fold), glutathione peroxidase (GPX1, 2.3-fold), GPX4 (1.6-fold), methionine sulfoxide reductase A (MSRA, 1.5-fold), peroxiredoxin 5 (PRDX5, 1.5-fold), PRDX6 (1.4-fold), thioredoxin 1 (TXN1, 1.6-fold), and catalase (2-fold) (Figure 3B). In contrast, only SOD2 and GPX1 were significantly upregulated in the *Mlkl^−/−^* HFHFrHC group. On the other hand, the partial loss of MLKL during the HFHFrHC feeding restored the levels of the mitochondrial antioxidant enzymes to those observed in the WT HFHFrHC group. While these data provide valuable insights into oxidative stress based on antioxidant enzyme activity, future research will consider supplementing this approach with other markers of oxidative stress to obtain a more comprehensive assessment. Thus, the diminished fatty acid metabolism in the HFHFrHC diet-fed *Mlkl^+/−^* mice was associated with decreased antioxidant enzymes, suggestive of reduced oxidative stress. Collectively, these data indicate that under the HFHFrHC diet conditions, the absence or partial reduction of *Mlkl* has distinct effects on the lipid droplet morphology and fat metabolism in the liver. Accordingly, only the partial loss of MLKL conferred protection against the HFHFrHC diet-induced liver damage and triglyceride accumulation.

### 2.3. Absence of Mlkl Exacerbated Liver Inflammation and Had No Impact on HFHFrHC Diet-Induced Liver Fibrosis

**Figure 4:**
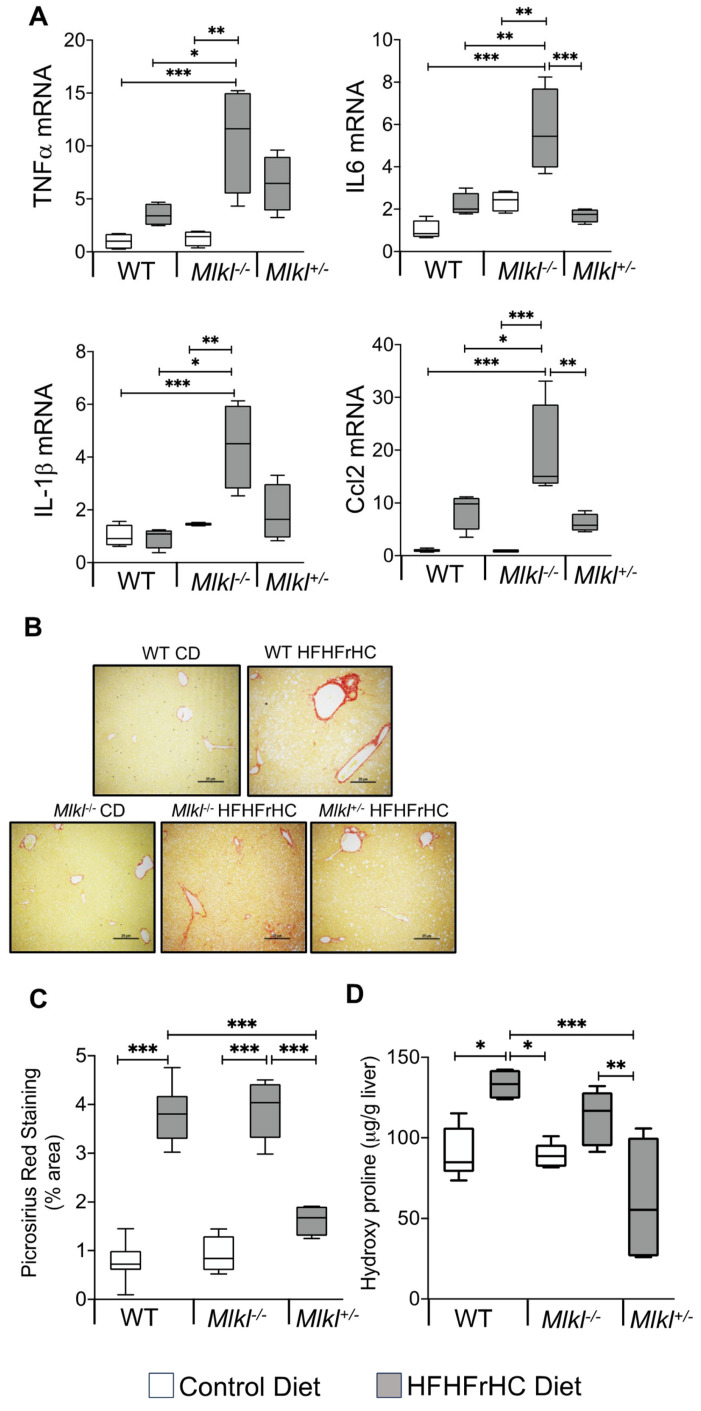
*Mlkl* deficiency exacerbated liver inflammation and had no impact on HFHFrHC diet-induced liver fibrosis. Data from WT, *Mlkl^−/−^* or *Mlkl^+/−^* mice fed a CD (white box plots) or HFHFrHC diet (box plots). (**A**) Transcript levels of the inflammatory cytokines TNFα, IL6, IL1β, and Ccl2, normalized to β-microglobulin and represented as the fold change relative to CD fed WT mice (*n* = 7–8 for WT; 5–8 for *Mlkl^−/−^* or *Mlkl^+/−^*). (**B**) Representative PSR staining of liver sections. Scale bar: 50 μM. (**C**) Quantification of PSR staining, represented as the percentage area (*n* = 3 per group). (**D**) Estimation of the total hydroxyproline content (µg/g) in the liver (*n* = 7–8 for WT; 5–8 for *Mlkl^−/−^* or *Mlkl^+/−^*). Error bars are represented as mean ± SEM. One-way ANOVA *p* < 0.05, * *p* < 0.05, ** *p* < 0.005, *** *p* < 0.0005.

We next investigated the impact of the absence or reduction of *Mlkl* on proinflammatory cytokines and chemokines in the liver because of the association of necroptosis with increased liver inflammation [19,31], and the documented reduction in proinflammatory cytokines in mice with *Mlkl* deficiency on a Western diet [21]. The RNA expression of 84 proinflammatory cytokines and chemokines in the livers of WT, *Mlkl^−/−^*, and *Mlkl^+/−^* mice subjected to either a CD or HFHFrHC diet were quantified. The detailed data from the mouse cytokines and chemokines array are provided in Table S2. Feeding the HFHFrHC diet significantly upregulated eight proinflammatory cytokines/chemokines in WT mice, and the top five genes elevated were Ccl22 (42-fold), IL-1rn (16-fold), IL-12b (15-fold), Ifna2 (12-fold), and IL-11 (5-fold) (Table S2). Interestingly, *Mlkl^−/−^* mice on the HFHFrHC diet significantly elevated 44 proinflammatory cytokine/chemokines in the liver when compared to WT mice on CD, and the top 5 genes upregulated were Spp1 (53-fold), Ccl22 (46-fold), Lif (35-fold), Ccl11 (33-fold), and IL-1rn (21-fold). Importantly, the necroptosis-associated proinflammatory cytokines TNFα (11-fold), IL6 (5.7-fold), IL-1β (4.6-fold), or CCL2 (15-fold) were also increased in the livers of *Mlkl^−/−^* fed the HFHFrHC diet (Figure 4A, Table S2). *Mlkl^+/−^* mice fed the HFHFrHC diet significantly upregulated 17 proinflammatory cytokine/chemokine genes, and the top 5 genes were Ccl22 (96-fold), Xcl1 (36-fold), IL-12b (20-fold), Ifna2 (15-fold), and Csf3 (2-fold). Overall, unexpectedly, the absence of *Mlkl* increased the proinflammatory cytokines and chemokines in the livers of HFHFrHC-fed mice.

Hepatocyte injury, inflammation, and the innate immune system promote liver fibrosis through the activation of hepatic stellate cells and the secretion and deposition of extracellular matrix [32]. Assessment of liver fibrosis revealed a significant increase in collagen fibers in the livers of WT mice fed the HFHFrHC diet, as measured by PSR staining (3 to 3.5-fold) or hydroxyproline content (1.5 to 2-fold), compared to those on the CD (Figure 4B–D). While the absence of *Mlkl* did not attenuate liver fibrosis induced by the HFHFrHC diet, *Mlkl^+/−^* mice exhibited a significant reduction in fibrosis. Collectively, these data suggest that while the absence of *Mlkl* did not protect against liver fibrosis induced by the HFHFrHC diet, the partial loss of *Mlkl* is protective.

To further confirm the inability of *Mlkl^−/−^* mice to protect against liver fibrosis, we used a methionine choline-deficient (MCD) diet, which is a widely used diet that induces fibrosis without obesity [33,34]. Our data in Figure S3A–C show that feeding an MCD diet resulted in comparable levels of liver fibrosis markers [PSR staining (6–6.5-fold), hydroxyproline levels (1.8-fold), and fibrosis markers (Col1α1, 4–5-fold and Col3α1, 2–2.5-fold)] in WT and *Mlkl^−/−^* mice compared to mice fed a methionine choline-sufficient diet (MSD). The transcript levels of TNFα, IL6, and IL1β were similar in the MCD diet-fed WT and *Mlkl^−/−^* mice, whereas Ccl2 levels were further increased by the absence of *Mlkl* (Figure S3D). Thus, the absence of *Mlkl* is not protective against diet-induced liver fibrosis.

### 2.4. Mlkl^−/−^ and Mlkl^+/−^ Mice Exhibited Reduced Adiposity in Response to HFHFrHC Diet

**Figure 5:**
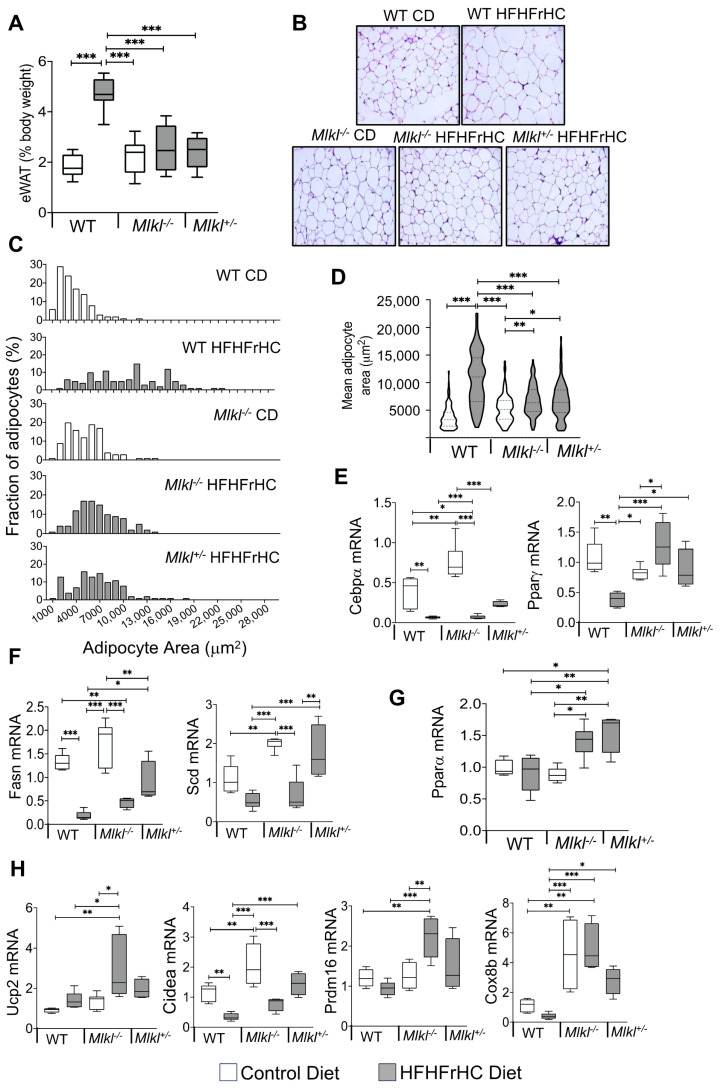
*Mlkl^−/−^* and *Mlkl^+/−^* mice exhibited reduced adiposity in response to HFHFrHC diet feeding. Data from WT, *Mlkl^−/−^* or *Mlkl^+/−^* mice fed a CD (white box plots) or HFHFrHC diet (box plots). (**A**) Percentage of epididymal white adipose tissue (eWAT) weight normalized to the body weight (*n* = 5–8 per/group). (**B**) H&E-stained sections of eWAT (magnification 20×). (**C**) Frequency distribution of adipocytes based on their size in eWAT (*n* = 3/group). (**D**) Mean surface area of adipocytes in eWAT (*n* = 3/group); transcript levels of proteins involved adipogenesis (Cebpα, Pparγ) (**E**), de novo lipogenesis (Fasn, Scd) (**F**), Pparα (**G**), and beiging (Ucp2, Cidea, Prdm16, Cox8b), normalized to β-microglobulin (**H**) (*n* = 7–8 for WT; 5–8 for *Mlkl^−/−^* or *Mlkl^+/−^*). Error bars are represented as mean ± SEM. One-way ANOVA *p* < 0.05, * *p* < 0.05, ** *p* < 0.005, *** *p* < 0.0005.

Contrary to the differential effects on the liver, both *Mlkl^−/−^* and *Mlkl^+/−^* mice on the HFHFrHC diet showed similar resistance to diet-induced fat accumulation (Figure 1C). Consistent with their body composition data, feeding the HFHFrHC diet resulted in a significant increase (2.5-fold) in the epididymal white adipose tissue (eWAT) deposition, normalized to the body weight, of WT mice but not for either *Mlkl^−/−^* or *Mlkl^+/−^* mice (Figure 5A). Histological examination of eWAT showed that WT mice on the HFHFrHC diet had different adipose morphologies, and adipocyte cellularity analysis revealed that the HFHFrHC diet fed *Mlkl^−/−^* and *Mlkl^+/−^* mice had significantly smaller adipocyte size distributions (Figure 5B,C). Moreover, the average adipocyte area shifted toward smaller adipocytes in *Mlkl^−/−^* and *Mlkl^+/−^* mice fed the HFHFrHC diet compared to WT mice on the same diet (Figure 5D). Next, we examined whether the decreased adipocyte size in *Mlkl^−/−^* or *Mlkl^+/−^* mice fed the HFHFrHC diet was due to impairments in either adipocyte differentiation or de novo lipogenesis. The absence of MLKL did not appear to impair the expression of the key adipocyte differentiation regulators CCAAT enhancer-binding protein alpha (Cebpα) or peroxisome proliferator-activated receptor gamma (Pparγ) in the eWAT of *Mlkl^−/−^* and *Mlkl^−/−^* mice on CD. In response to the HFHFrHC diet, Cebpα and Pparγ were suppressed in the WT group but significantly increased in both the *Mlkl^−/−^* and *Mlkl^+/−^* groups relative to the WT HFHFrHC diet fed mice (Figure 5E). Analysis of the de novo lipogenesis markers (fatty acid synthase (Fasn) and stearoyl-CoA desaturase (Scd)) revealed that the absence of MLKL (*Mlkl^−/−^* mice) significantly elevated Scd levels but not FASN on a CD, whereas feeding the HFHFrHC diet significantly reduced the levels of Fasn, but not SCD in either WT or *Mlkl^−/−^* mice. In contrast, both Fasn and Scd were significantly upregulated in *Mlkl^+/−^* mice (Figure 5F). Analysis of the transcript levels of the proinflammatory cytokines/chemokines showed that feeding the HFHFrHC diet increased the level of Ccl2 (2.5-fold), but not TNFα, IL6, or IL1β, in the eWAT of WT mice, and the Ccl2 levels were significantly downregulated in *Mlkl^−/−^* and *Mlkl^+/−^* mice fed the HFHFrHC diet (Figure S4A–D). These results suggest that the improved adipocyte cellularity sizes of the *Mlkl^−/−^* or *Mlkl^+/−^* mice during the HFHFrHC diet feeding is due to enhanced expression of adipogenic regulators Cebpα and Pparγ.

Next, we tested whether the level of Pparα, a key regulator of lipid metabolism and energy expenditure in adipose tissue, is altered in eWAT [35]. The HFHFrHC diet did not alter the Pparα levels in the eWAT of WT mice, whereas Pparα was significantly upregulated in *Mlkl^−/−^ and Mlkl^+/−^* mice (Figure 5G). Given the fact that Pparγ and Pparα synergize to induce WAT browning/beiging to enhance energy expenditure [35], we evaluated the impact of MLKL reduction or absence on the browning markers (uncoupling protein 2 (Ucp2), PR/SET domain 16 (Prdm16), cell death inducing DFFA like effector a (Cidea), and Cox8b cytochrome c oxidase subunit 8B (Cox8b)) in eWAT. The absence of *Mlkl* had no effect on the Ucp2 and Prdm16 levels, while Cidea and Cox8b were significantly upregulated compared to WT mice fed CD. Feeding the HFHFrHC diet significantly upregulated the Ucp2, Prdm16, and Cox8b levels in *Mlkl^−/−^* mice compared to WT mice. In *Mlkl^+/−^* mice fed the HFHFrHC diet, Cidea and Cox8b were significantly upregulated compared to WT mice, while Ucp2 and Prdm16 showed a tendency for an increase without reaching statistical significance (Figure 5H). Collectively, the upregulation of beige adipocyte genes in combination with the smaller adipocyte size distributions suggests a potential link between MLKL absence/reduction and improved insulin sensitivity in the animal, with browning of the WAT contributing to reduced adiposity.

### 2.5. Mlkl^−/−^ and Mlkl^+/−^ Mice Exhibited Improved Insulin Sensitivity on a HFHFrHC Diet

**Figure 6:**
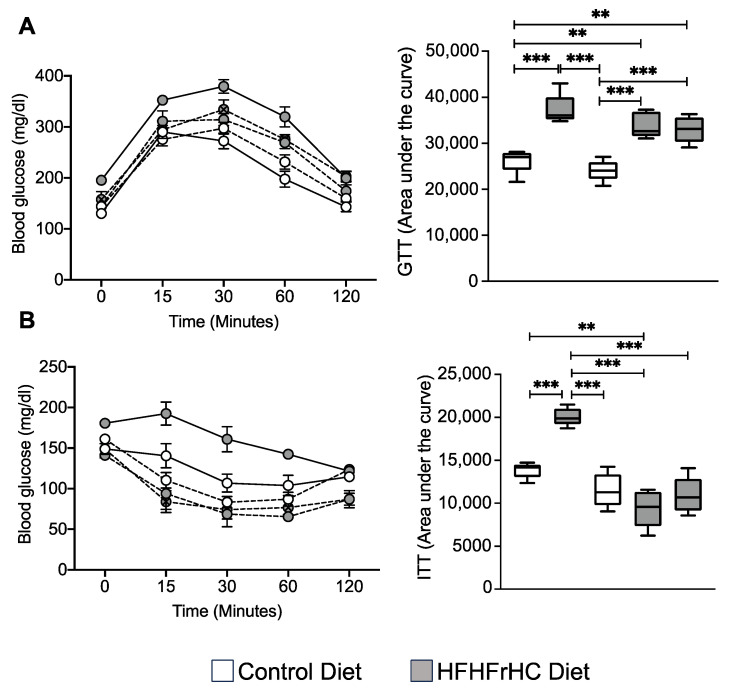
*Mlkl^−/−^* and *Mlkl^+/−^* mice exhibited improved insulin sensitivity on a HFHFrHC diet. Data from WT, *Mlkl^−/−^* or *Mlkl^+/−^* mice fed a CD (white circles/box plots) or HFHFrHC diet (gray circles/box plots). (**A**) Glucose tolerance test (left panel) and area under the curves (AUCs) for GTT (right panel). (**B**) Glucose tolerance test (left panel) and area under the curves (AUCs) for ITT (right panel), (*n* = 7–8 for WT; 5–8 for *Mlkl^−/−^* or *Mlkl^+/−^*). One-way ANOVA *p* < 0.05, ** *p* < 0.005, *** *p* < 0.0005.

Reduced adiposity, as well as a smaller but more adipocyte cellularity, is associated with enhanced insulin sensitivity [36,37]. We performed the glucose tolerance test (GTT) to assess the efficiency of clearance following a bolus of glucose in the WT, *Mlkl^−/−^* and *Mlkl^+/−^* groups provided a CD or HFHFrHC diet. Consumption of the HFHFrHC diet led to comparable levels of glucose clearance in WT, *Mlkl^−/−^*, and *Mlkl^+/−^* mice and no difference in the area under the curve was observed (Figure 6A). Next, insulin sensitivity was evaluated using the insulin tolerance test (ITT) to assess how efficiently glucose is cleared from circulation following administration of insulin. The HFHFrHC diet-feeding induced insulin resistance in WT mice, whereas *Mlkl^−/−^* and *Mlkl^+/−^* mice displayed significantly improved insulin sensitivity compared to WT mice on the same diet (Figure 6B). Together, these findings suggest that the loss of MLKL does not improve the body’s ability to handle a glucose load; rather, MKLK-deficient mice have an increase in peripheral insulin sensitivity.

### 2.6. Energy Expenditure Is Increased in Mlkl^−/−^ and Mlkl^+/−^ Mice on an HFHFrHC Diet

**Figure 7:**
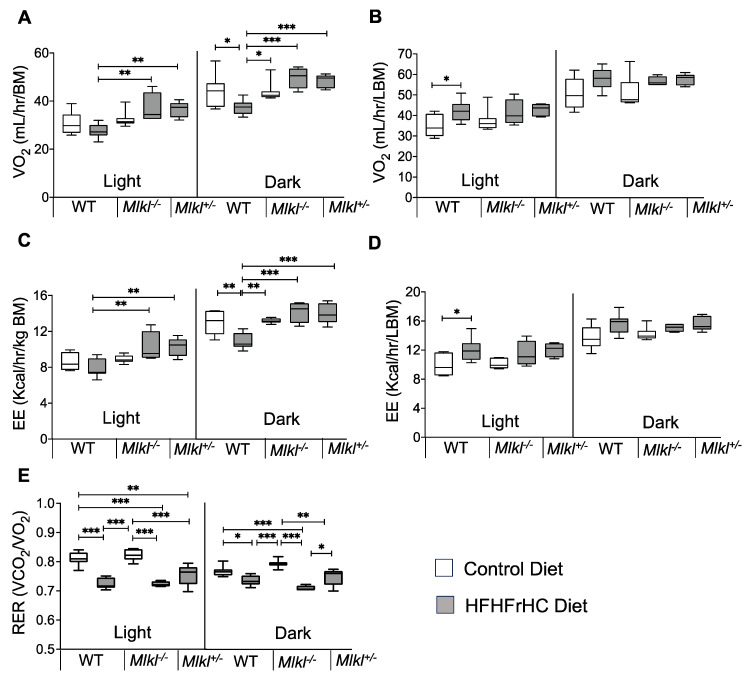
*Mlkl^−/−^* and *Mlkl^+/−^* mice exhibited reduced adiposity in response to the HFHFrHC diet. Indirect calorimetric data from WT, *Mlkl^−/−^* or *Mlkl^+/−^* mice fed a CD (white box plots) or HFHFrHC diet (box plots). Whole-body oxygen consumption rate normalized to the total body mass (**A**) or lean body mass (**B**). Average energy expenditure normalized to the total body mass (**C**) or lean body mass (**D**). (**E**) Average RER (VCO_2_/VO_2_). Error bars are represented as mean ± SEM. One-way ANOVA *p* < 0.05, * *p* < 0.05, ** *p* < 0.005, *** *p* < 0.0005.

We performed indirect calorimetric analysis to further evaluate the role of MLKL in whole body metabolism. The HFHFrHC diet significantly reduced oxygen consumption in WT mice when normalized to the body mass during the dark phase compared to CD-fed WT mice (Figure 7A and Figure S5A). Conversely, *Mlkl^−/−^* and *Mlkl^+/−^* mice on the HFHFrHC diet exhibited increased oxygen consumption during the light (20%, 30%, respectively) and dark (50%, 50%, respectively) phases compared to WT mice on the same diet. Additionally, energy expenditure (EE) normalized to the total body mass was elevated in HFHFrHC diet-fed *Mlkl^−/−^* and *Mlkl^+/−^* mice during both light (12%, 22%, respectively) and dark (40%, 25%, respectively) phases compared to WT mice on the same diet (Figure 7C and Figure S5C). Remarkably, when normalized to the lean body mass, oxygen consumption and EE appeared comparable among the HFHFrHC diet-fed WT, *Mlkl^−/−^*, and *Mlkl^+/−^* mice, suggesting a role of adipose tissue in these processes (Figure 7B,D and Figure S5B,D). The Respiratory Exchange Ratio (RER) measurements, reflecting metabolic substrate utilization [38], demonstrated significantly reduced RER values (near 0.7) for the HFHFrHC diet-fed groups compared to the CD-fed groups, indicating that fat is used as the major substrate by WT, *Mlkl^−/−^*, and *Mlkl^+/−^* mice during both light and dark phases when the mice are fed the HFHFrHC diet (Figure 7E and Figure S5E). When comparing the RER values of HFHFrHC-fed *Mlkl^−/−^* and *Mlkl^+/−^* mice in the dark phase, *Mlkl^+/−^* mice exhibited a significantly higher RER value compared to *Mlkl^−/−^* mice, indicating a potential utilization of both fat and carbohydrate sources (Figure 7E).

## 3. Discussion

Necroptosis, a highly proinflammatory mode of cell death, is elevated in MAFLD/MASH patients and mouse models, and inflammation is a key driver of the progression of MAFLD [12,14,39]. Nevertheless, studies investigating the consequences of inhibiting necroptosis through targeting either RIPK3 or MLKL in mice have presented contradictory outcomes concerning inflammation, steatosis, and fibrosis, with the observed effects varying based on the dietary conditions [40,41,42,43]. Hence, the contribution of necroptosis to the onset and advancement of MAFLD remains uncertain. The results of our study imply that while the absence of *Mlkl* (*Mlkl^−/−^* mice) is protective against HFHFrHC diet-induced obesity and insulin resistance, it does not extend the same protection to the liver against inflammation, steatosis or fibrosis. In contrast, the partial loss of *Mlkl* (*Mlkl^+/−^* mice) mitigated the adverse effects of an HFHFrHC diet on both adipose tissue and the liver, while also enhancing insulin sensitivity.

Current evidence supports a role of necroptosis and necroptosis-induced inflammation in the progression of MAFLD [18,23,39,44]. Contrary to these findings, we did not find an increase in the necroptosis markers or necroptosis-associated proinflammatory cytokines (TNFα, IL6, IL-1β, and CCL2) in the livers of WT mice fed the HFHFrHC diet, despite observing an increase in the MLKL protein expression. Our results align with those of Xu et al., who observed no elevation in the necroptosis-related inflammatory cytokines in a MAFLD mouse model induced by a high-fat diet. Nonetheless, they did report an upsurge in the necroptosis markers [22]. Notably, our investigation demonstrates that the HFHFrHC diet diminishes the expression of RIPK3, a critical initiator of MLKL activation via phosphorylation. This implies a potential involvement of reduced RIPK3 expression in inhibiting necroptosis. In support of this, a recent study reported epigenetic silencing of *Ripk3* in hepatocytes of a MASH-inducing diet mouse model [45]. Liver cancer cells are also shown to prevent MLKL-mediated necroptosis by epigenetic silencing of *Ripk3* [46]. Surprisingly, in our study, *Mlkl^−/−^* mice on the HFHFrHC diet exhibited elevated levels of TNFα, IL6, IL1β, and Ccl2 in the liver, challenging previous data indicating that MLKL deficiency reduces inflammation [23,44]. However, this agrees with reports linking MLKL deficiency to increased proinflammatory cytokines due to the non-necroptotic function of MLKL in regulating the intracellular degradation of TNF and its receptor [47]. While MLKL is traditionally viewed as operating downstream of RIPK3 in necroptosis, recent reports suggest potential necroptosis-independent functions for MLKL, such as autophagy, receptor internalization, ligand-receptor degradation, endosomal trafficking, extracellular vesicle generation, neutrophil trap formation, and inflammasome control [48]. Moreover, the specific functions of MLKL in different cell types could also influence liver inflammation and pathology. A recent study highlighted that the absence of *Mlkl* specifically in myeloid cells exacerbated ethanol-induced hepatic injury, steatosis, and inflammation in mice [49]. Therefore, further research is needed to explore the impact of the cell type-specific effects of MLKL on MASH, underscoring the intricate nature of MLKL’s involvement in liver pathology and expanding its potential functions beyond conventional associations with necroptosis.

In our study, we used the HFHFrHC diet because it is widely used to induce liver fibrosis and exhibits several clinically relevant characteristics of MASH and associated metabolic disorders in mice [24]. Whereas *Mlkl^−/−^* mice did not show protection against HFHFrHC diet-induced liver damage, steatosis, or fibrosis, the partial loss of *Mlkl* (*Mlkl^+/−^* mice) is protective. These findings are consistent with our previous data showing that the absence of *Mlkl* does not protect mice against liver fibrosis induced by a choline-deficient high-fat diet [23]. Therefore, the reported protection of mice from CCl_4_-induced or bile duct ligation-mediated liver fibrosis in the absence of *Mlkl* suggests a diet-specific influence on fibrosis distinct from the injury mediated by CCl_4_ or bile duct ligation [50,51]. The discovery that knocking out *Mlkl* specifically in endothelial cells alleviates liver fibrosis in a mouse MASH model underscores the importance of comprehending the cell-type-specific effects of MLKL in order to discern its role in MASH [52]. The absence of MLKL altered the lipid droplet size and distribution without affecting the overall triglyceride accumulation, suggesting a potential role of MLKL in regulating the lipid droplet size. The protective effect observed of the *Mlkl^+/−^* mice could be linked to increased levels of Pparα and Pparγ in the liver, which are critical regulators of liver lipid metabolism and fibrosis, respectively [53,54]. The fact that the Pparα and Pparγ levels are increased only in the HFHFrHC diet-fed *Mlkl^+/−^* mice, and not in WT or *Mlkl^−/−^* mice fed the same diet, hints at a dose-dependent relationship between MLKL and PPARs, necessitating further investigation into the underlying mechanisms of this novel MLKL–PPAR connection in MAFLD.

A key finding of our study is the resistance to diet-induced obesity in both *Mlkl^−/−^* and *Mlkl^+/−^* mice, consistent with similar effects reported in *Mlkl^−/−^* mice with other MAFLD-inducing diets [21,22]. Our findings show that the resistance to obesity is not due to the result of alterations in food intake or physical activity but appears to be due to an impact of *Mlkl* on adiposity. The presence of smaller adipocytes in the HFHFrHC diet-fed *Mlkl^−/−^* and *Mlkl^+/−^* is not due to impaired adipogenesis or de novo lipogenesis; rather, it is due to increased fat utilization. Increased fat utilization in these mouse models is further supported by the elevated Ppar*α* levels and browning markers in white adipose tissue [35]. The increased oxygen consumption and energy expenditure in both *Mlkl^−/−^* and *Mlkl^+/−^* mice is associated with fat tissue, suggesting a role for MLKL in influencing fat utilization in adipose tissue. Thus, increased fat utilization by thermogenic adipocytes in the absence or reduction of MLKL contributes to the resistance to diet-induced obesity observed in these mouse models. While our data contradict previous cell culture studies using 3T3-L1 cells, which showed that knocking down *Mlkl* impairs white adipocyte differentiation and downregulates genes involved in lipid metabolism [55], our findings in vivo could suggest the involvement of other factors, such as adipose tissue extracellular matrix or systemic effects on adipocyte differentiation, in the absence or partial loss of *Mlkl* [56,57,58]. The fact that adipose tissue inflammation is not affected by an HFHFrHC diet or MLKL supports a non-necroptotic function of MLKL in regulating lipid metabolism in adipose tissue, which warrants further investigation for potential therapeutic targets in obesity-related conditions.

The improved insulin sensitivity observed in the HFHFrHC diet-fed *Mlkl^−/−^* and *Mlkl^+/−^* mice, as evidenced by insulin tolerance tests, suggests a potential role of MLKL in the regulation of systemic insulin responsiveness. However, it is noteworthy that the glucose tolerance remained unaltered in these mice. This observation implies that while MLKL deficiency might have specific effects on insulin-mediated glucose clearance, it might not significantly impact overall glucose homeostasis. In agreement with our results, Xu et al. [22] documented enhanced insulin sensitivity in *Mlkl^−/−^* mice on an HFD. However, diverging from our findings, they also noted improved glucose tolerance. Again, this inconsistency suggests diet as a critical factor that may contribute to the observed varied outcomes. Pparγ is a key regulator of adipocyte differentiation and function that is known to regulate systemic insulin sensitivity [26]. Thus, the upregulation of Pparγ in the context of MLKL deficiency or reduction may contribute to the observed improvements in insulin sensitivity in our study.

In our study, to induce MASH, we used a diet that contains 40 kcal% fat (mostly palm oil), 20 kcal% fructose, and 2% cholesterol. Palm oil is rich in saturated fats and is shown to induce steatosis, hepatocyte ballooning, and MAFLD in mice compared to mice fed a olive oil-rich diet, which underscores the potentially significant adverse effects of its overconsumption due to the food industry’s heavy reliance on palm oil [59]. Excessive fructose consumption is known to favor MAFLD, as fructose is both a substrate and an inducer of hepatic de novo lipogenesis [60]. The excessive intake of fructose-containing beverages has particularly strong links to childhood obesity and pediatric NAFLD. Moreover, in adults aged 48 years and older, daily fructose consumption has been shown to elevate hepatic inflammation and hepatocyte ballooning [61]. Similarly, a high-cholesterol diet exacerbates liver fibrosis in mice [62] and dietary cholesterol drives progression of MAFLD to HCC in mice [63]. Overall, these dietary components may interact synergistically to promote the development and progression of MAFLD, highlighting the importance of considering diet composition in preclinical studies of liver disease.

### Limitations and Conclusions

This is the first study to examine the effect of *Mlkl* complete or partial deletion on diet-induced MASH. There are a couple of limitations to our study. First, we acknowledge the potential limitation of not including an *Mlkl^+/−^* group fed a control diet. This decision was based on our preliminary studies, which indicated that WT and *Mlkl^+/−^* mice fed a control diet did not demonstrate any phenotypic changes. Nevertheless, our primary aim in this study was to assess how the absence or partial reduction of *Mlkl* affects liver pathology and metabolic parameters in response to an obesity-inducing diet. Second, the experiments involving diet-induced obesity were conducted under standard housing conditions with temperatures ranging from 20 to 23 °C, which poses a mild cold challenge for laboratory mice. The mild cold challenge experienced by mice in standard housing conditions significantly increases energy expenditure, potentially influencing metabolic responses to high-calorie diets and obscuring interpretations of obesity development. Whether thermoneutral conditions will exert a similar effect on the obesity phenotype is not explored in the current study [64]. Finally, despite observing improved insulin sensitivity in both *Mlkl^−/−^* and *Mlkl^+/−^* mice, we did not investigate the specific peripheral tissues, such as skeletal muscle, liver, or adipose tissue, to identify the underlying mechanisms contributing to their enhanced metabolic health.

In summary, our study underscores the diverse and non-necroptotic roles of MLKL in two key metabolic tissues, liver and white adipose tissue. Our findings challenge prior studies regarding MLKL’s involvement in liver inflammation in diet-induced MAFLD and the role of necroptosis-induced liver inflammation in fibrosis. Additionally, this study emphasizes the importance of a dose-dependent relationship of MLKL in the liver during MAFLD. Overall, the research sheds light on the intricate interplay between MLKL, lipid metabolism, and metabolic health, offering valuable insights for understanding and potentially targeting MLKL in the context of obesity-related disorders. Furthermore, cell type-specific and mechanistic studies are warranted to elucidate the precise pathways through which MLKL exerts its influence on these physiological processes. The translational implications of these findings may offer new avenues for therapeutic interventions in conditions characterized by metabolic dysfunction and liver pathology.

## 4. Materials and Methods

### 4.1. Animals and Diet Feeding

All the procedures were approved by the Institutional Animal Care and Use Committee at the University of Oklahoma Health Sciences Center (OUHSC). *Mlkl^−/−^* mice were developed by Murphy et al. (2013) [65], and in our study, colonies of WT (*Mlkl^+/+^*)*, Mlkl^−/−^* and *Mlkl^+/−^* mice were generated by breeding male and female *Mlkl^+/−^* mice. The WT (*Mlkl^+/+^*) mice used in the study were littermates from *Mlkl^+/−^* mice cross-breeding. The mice were group housed in ventilated cages at 20  ±  2 °C, 12 h/12 h dark/light cycle. Diet-feeding was performed for 6 months, starting at 2 months of age. Male mice (*n* = 5–8/group) were fed either a high-fat, high-fructose, high cholesterol diet (HFHFrHC) containing 40 kcal% fat (mostly palm oil), 20 kcal% fructose, and 2% cholesterol (D09100310, Research Diets Inc, New Brunswick, NJ, USA) or a matched control diet (D09100304, Research Diets Inc, New Brunswick, NJ, USA) ad libitum for a period of 6 months at the OUHSC animal care facility. The detailed diet composition is presented in Supplementary Table S3 and the experimental design is presented in Figure 1A. At the end of the study, mice were euthanized by isoflurane inhalation followed by cervical dislocation as a secondary method, and all the tissues were immediately collected, flash frozen in liquid nitrogen, and stored at −80 °C until further analyses.

### 4.2. Glucose Tolerance and Insulin Tolerance Tests (GTTs and ITTs)

GTTs and ITTs were performed as described before [66]. In brief, mice were fasted for 6 h (for the GTT) or 5 h (for the ITT) and received an intraperitoneal injection of glucose (1 g/kg, Sigma Aldrich, St. Louis, MO, USA) or insulin (0.75 units/kg, Novo Nordisk Inc., Bagsvaerd, Denmark). The glucose concentration was determined from blood samples collected from the tail vein after 15, 30, 60 and 120 min of glucose or insulin challenge using TRUE METRIX glucose strips and glucometer (Trividia Health Inc., Plainsboro Township, NJ, USA).

### 4.3. Histological Analysis of Liver Sections

Paraffin-embedded liver or visceral fat sections were stained with Hematoxylin & Eosin (H&E) according to the standardized protocol at the Stephenson Cancer Center Tissue Pathology core. Images were acquired using a Nikon Ti Eclipse microscope (Nikon, Melville, NY, USA) at 20× magnification for 3 random non-overlapping fields per sample. The lipid droplet number and area were quantified using Adiposoft (v1.16) Fiji (v2.15.0) extension software as described in [67] and are represented graphically.

### 4.4. Picrosirius Red Staining

Picrosirius red staining was performed with paraffin-embedded liver sections (4 µM) by following a standardized protocol at the Imaging Core facility at the Stephenson Cancer Center Tissue Pathology Core. Images were acquired using a Nikon Ti Eclipse microscope (Nikon, Melville, NY, USA) for 3 random non-overlapping fields per sample at 20× magnification and quantified using Image J software (v1.54g) (U.S. National Institutes of Health).

### 4.5. Alanine Transaminase (ALT) and Triglyceride Assessment

The plasma ALT levels, plasma and hepatic triglycerides were quantified using Alanine transaminase or Triglyceride colorimetric assay kits obtained from Cayman Chemical Company (Ann Arbor, MI, USA). The measurements were conducted in accordance with the manufacturer’s instructions.

### 4.6. Hydroxyproline Assay

The collagen content in the liver was measured using the hydroxyproline content as described in [23]. The absorbance values at 558 nm were converted into µg units using the 4-parameter standard curve generated using the standards and expressed as µg hydroxyproline/g of tissue.

### 4.7. Indirect Calorimetry Studies

Indirect calorimetry was performed using a 16-chamber metabolic monitoring system (Syble Systems Itnl., Promethion) for the continuous monitoring of CO_2_ production and O_2_ consumption in individual mice. Mice destined for metabolic monitoring were acclimated to the system for 48–72 h prior to the data collection periods. Following acclimation, mice were monitored continuously for 72 h, during which time the energy intake (EI) was measured in real time. The energy intake, respiratory exchange ratio (vCO_2_/vO_2_), energy expenditure and energy balance measurements were calculated as an average of the measures over the period of study. Activity was determined by infrared beam breaks as a measure of the horizontal and vertical movement (XYZ-axis) of mice. The food and water intake amount and the frequency/duration of feeding behavior within the 24–120 h experimental window was quantified by the Promethion system. The body composition was quantified by magnetic resonance with a 1 min primary accumulation time (4-in-1 700 EchoMRI, Houston, TX, USA).

### 4.8. Western Blot Analysis

Western blot analysis was performed and quantified using ImageJ software (v1.54g) (U.S. National Institutes of Health, Bethesda, MD, USA) as described previously [18]. The primary antibodies used were: MLKL (MABC60, Millipore, Burlington, MA, USA); P-MLKL (#ab196436, Abcam, Cambridge, UK); RIPK3 (#NBP1-77299, Novus Biologicals, Centennial, CO, USA); and β-tubulin (sc-5274, Santa Cruz Biotechnology, Dallas, TX, USA). In the representative Western blots, each experimental group is represented by 4 independent mice. Graphical representations of the quantified Western blots for 4 animals/group are shown alongside the blots.

### 4.9. Quantitative Real-Time PCR (RT-PCR)

Briefly, the total RNA was isolated using the RNeasy kit (Qiagen, Valencia, CA, USA), first-strand cDNA was synthesized using the High-capacity cDNA reverse transcription kit (Thermo Fisher Scientific, Waltham, MA, USA), and RT-PCR using Power SYBR Green PCR Master Mix (Thermo Fisher Scientific, Waltham, MA, USA) in a Quantstudio 12K Flex real-time PCR system (Applied Biosystems, Waltham, MA, USA). The primers used for the RT-PCR are listed in Table S1. The PCR arrays were performed using RT^2^ Profiler™ PCR Array Mouse Cytokines and Chemokines (PAMM-150Z, Qiagen, Venlo, The Netherlands). The calculations were performed by a comparative method (2^−ΔΔ^Ct) using β-microglobulin, β-actin, or hypoxanthine phosphoribosyltransferase 1 (HPRT) as controls, as described previously [23]. The expression of genes, comparison of signatures between the groups and heat map representation was performed using Microsoft Excel (Version 16.78.3, 2023) and MetaboAnalyst 5.0.

### 4.10. Quantitative Targeted Proteomics

Quantitative proteomics was used to determine the changes in mitochondrial enzymes in the liver tissue as previously described [18,68]. Briefly, 20 μg total liver tissue homogenate was run 1.5 cm into a 12.5% SDS–PAGE gel (Criterion, Bio-Rad, Berkeley, CA, USA). This was followed by fixation and staining with GelCode Blue (Pierce, Appleton, WI, USA). The entire lane was cut into ~1 mm^3^ pieces, washed, reduced with DTT, alkylated with iodoacetamide, and digested with trypsin. The peptides generated were extracted with 50% methanol/10% formic acid in water, dried, reconstituted in 1% acetic acid, and analyzed using selected reaction monitoring (SRM) with a triple quadrupole mass spectrometer (Thermo Scientific TSQ Quantiva, Waltham, MA, USA) configured with a splitless capillary column HPLC system (Thermo Scientific Ultimate 3000, Waltham, MA, USA). Data processing was performed using the program Skyline, which aligned the various collision-induced dissociation reactions monitored for each peptide and determined the chromatographic peak areas. The response for each protein was taken as the total response for all the peptides monitored. The changes in the relative abundance of the proteins were determined by normalization to the BSA internal standard, with confirmation by normalization to the housekeeping proteins.

### 4.11. Statistical Analysis

All the quantitative data are represented as the mean ± SEM. One-way ANOVA with Tukey’s multiple comparison test was used to analyze the data using GraphPad Prism (Version 10.0.3). *p* < 0.05 is considered statistically significant. The symbols used for the statistical comparison between groups are described in the figure legends.

## Data Availability

Data are contained within the article or Supplementary Materials.

## Supplementary Materials

The following supporting information can be downloaded at https://www.mdpi.com/article/10.3390/ijms25052813/s1.
