# Supplementary material for: Non-Necroptotic Roles of MLKL in Diet-Induced Obesity, Liver Pathology, and Insulin Sensitivity: Insights from a High-Fat, High-Fructose, High-Cholesterol Diet Mouse Model
Source: Int J Mol Sci. 2024 Feb 28;25(5):2813. doi: 10.3390/ijms25052813 (PMC10931720; doi:10.3390/ijms25052813)
Supplement: Supplementary file 1 [file ijms-25-02813-s001.zip › Supplementary Figures.pdf]

SUPPLEMENTARY FIGURES

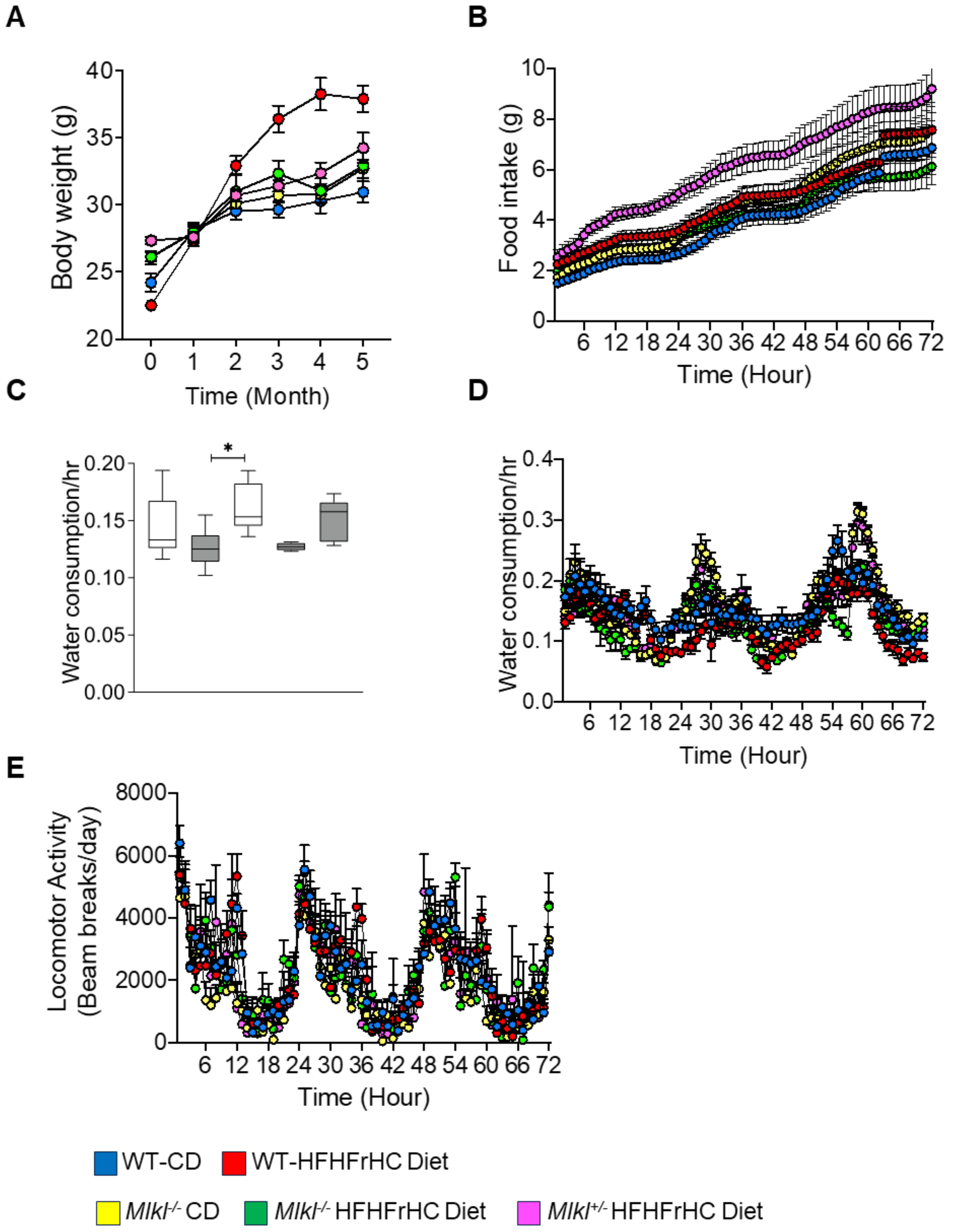

Figure S1: **Changes in body weight, food intake, water consumption, and locomotor activity of CD or HFHFrHC diet fed WT, *Mlkl*$^{-/-}$ or *Mlkl*$^{+/-}$ mice. (A)** Change in body weight during diet

feeding from baseline through six months; Indirect calorimetry measurements over a period of 72 hours after 6 months of diet feeding showing daily food intake **(B)**, average water consumption **(C)**, daily profile of water consumption **(D)**, and daily profile of spontaneous locomotor activity **(E)**. In the figure- Blue: WT mice on CD; Red: WT mice on HFHFrHC diet; Yellow: *Mlkl*$^{-/-}$ mice on CD; Green: *Mlkl*$^{-/-}$ mice on HFHFrHC diet and Pink: *Mlkl*$^{+/-}$ mice on HFHFrHC diet. (n= 7-8 for WT; 5-8 for *Mlkl*$^{-/-}$ or *Mlkl*$^{+/-}$). White and gray bars box plots represent experimental groups fed either CD or HFHFrHC diet, respectively. Error bars are represented as mean± SEM. One-way ANOVA P0.05, * p< 0.05, ** p< 0.005, ***p< 0.0005

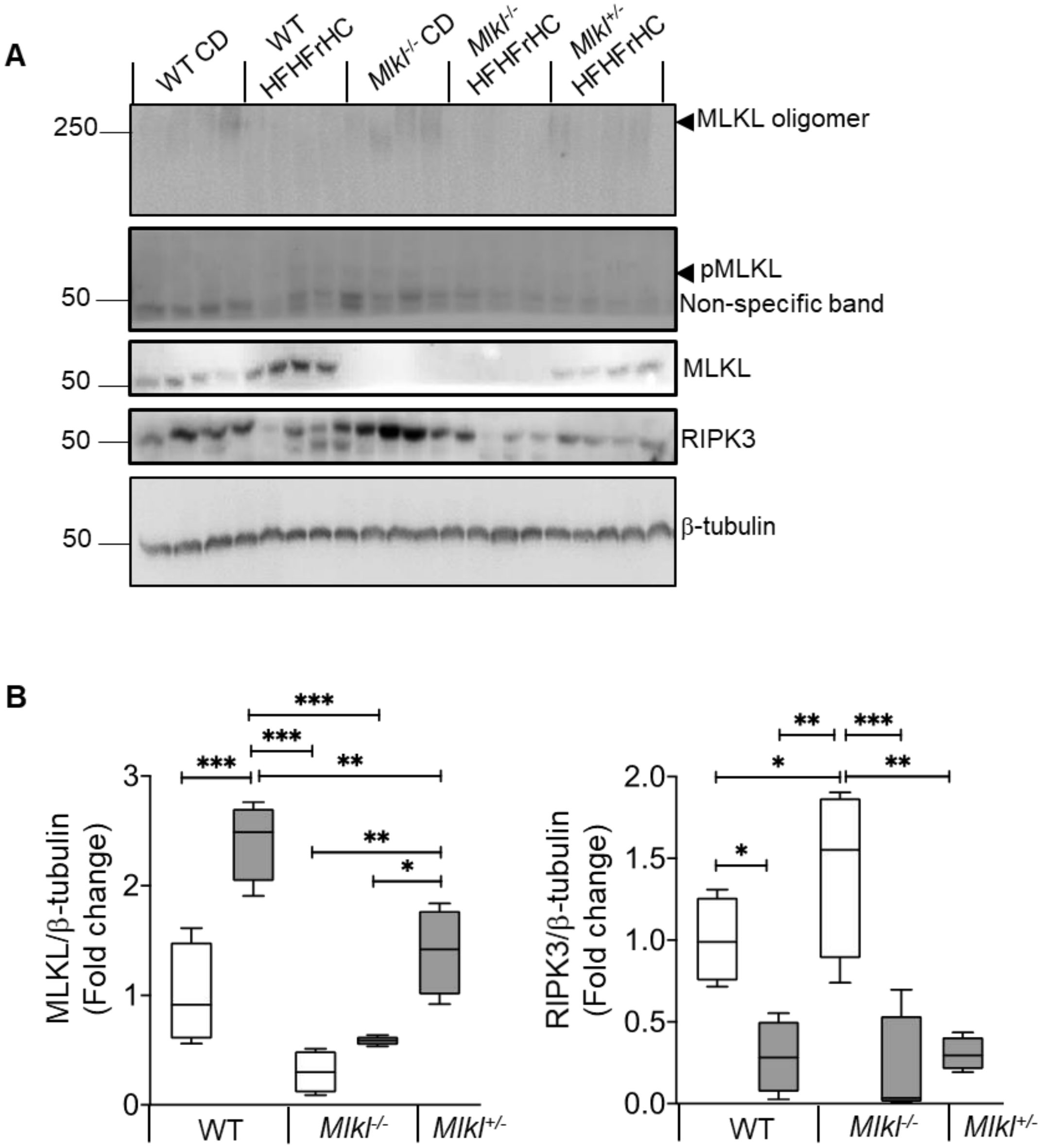

Figure S2: Necroptosis markers in the livers of CD or HFHFrHC diet fed mice. (A) Immunoblots of liver tissue extracts for necroptosis proteins: RIPK3, MLKL, pMLKL, MLKL oligomer, and loading control β-tubulin from WT, *Mlkl*$^{-/-}$ or *Mlkl*$^{+/-}$ fed test diets; **(B)** Graphical representation of quantified blot normalized to β-tubulin. White and gray bars box plots represent experimental groups fed either CD or HFHFrHC, respectively. Error bars are represented as mean± SEM. One-way ANOVA P0.05, * p< 0.05, ** p< 0.005, ***p< 0.0005

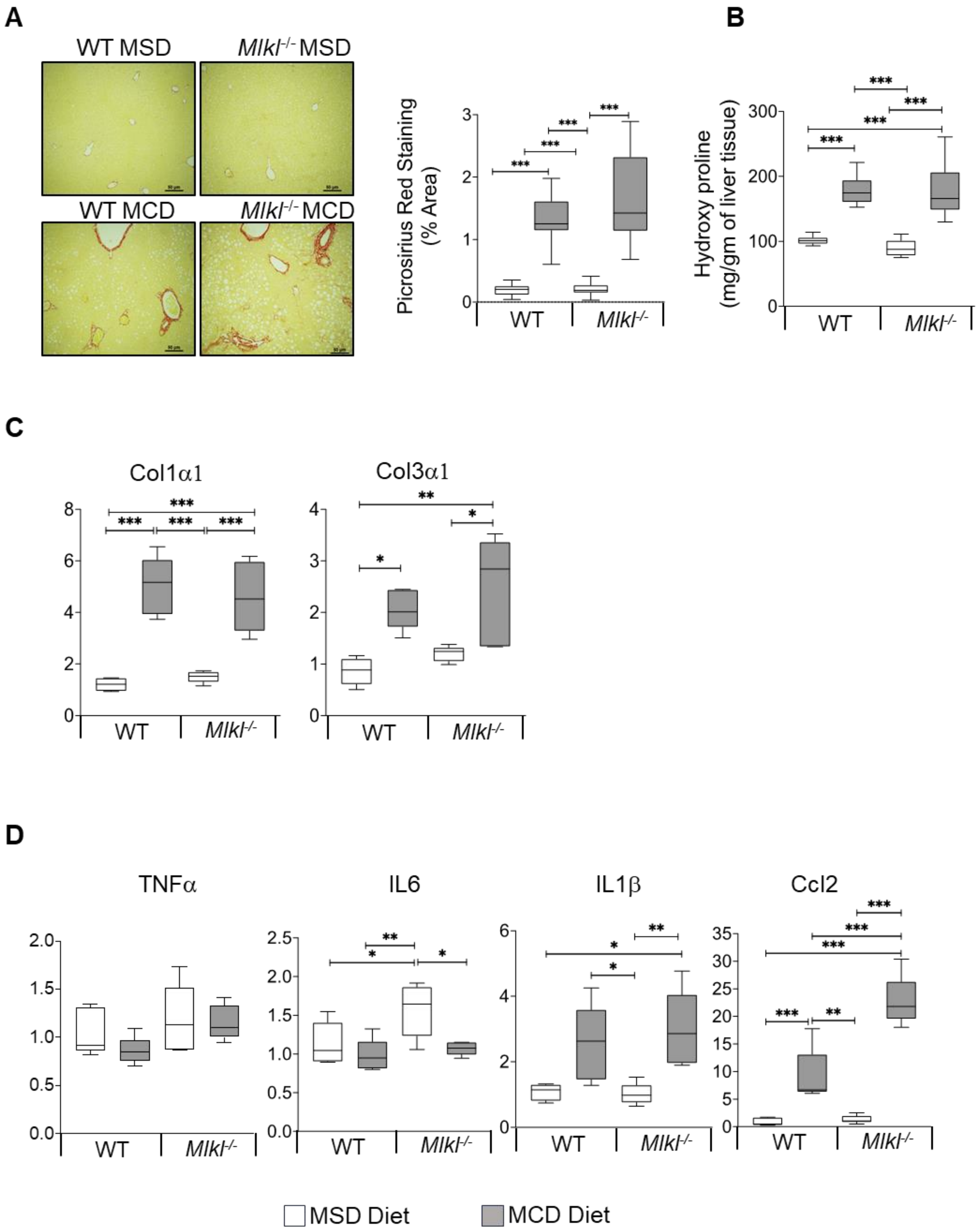
A
WT MSD
$Mlkl^{-/-}$ MSD
WT MCD
$Mlkl^{-/-}$ MCD
Picrosirius Red Staining (% Area)
WT
$Mlkl^{-/-}$
B
Hydroxy proline (mg/gm of liver tissue)
WT
$Mlkl^{-/-}$
C
Col1α1
Col3α1
WT
$Mlkl^{-/-}$
D
TNFα
IL6
IL1β
Ccl2
WT
$Mlkl^{-/-}$
MSD Diet
MCD Diet

Figure S3: Fibrosis markers in the livers of MSD or MCD diet fed mice. (A) *Left:* Representative PSR staining of liver sections of WT, *Mlkl*$^{-/-}$ fed either methionine-choline sufficient diet (MSD) or methionine-choline deficient (MCD) diet. Scale bar: 50 mM. *Right:* Graphical representation of PSR quantification. (n=3/group); **(B)** Estimation of total hydroxyproline content in liver tissues of WT, *Mlkl*$^{-/-}$ mice fed MSD or MCD (n=5/group); **(C)** The transcript levels fibrosis markers Col1a1 and Col3a1 and inflammatory cytokine markers TNFα, IL6, IL1β, and Ccl2 **(D)** in the livers of WT, *Mlkl*$^{-/-}$ fed MSD, or MCD. Each transcript is represented as a fold change normalized to β-microglobulin (n=6/group). White and gray bars box plots represent experimental groups fed either CD or HFHFrHC, respectively. Error bars are represented as mean± SEM. One-way ANOVA P0.05, * $p< 0.05$, ** $p< 0.005$, ***$p< 0.0005$.

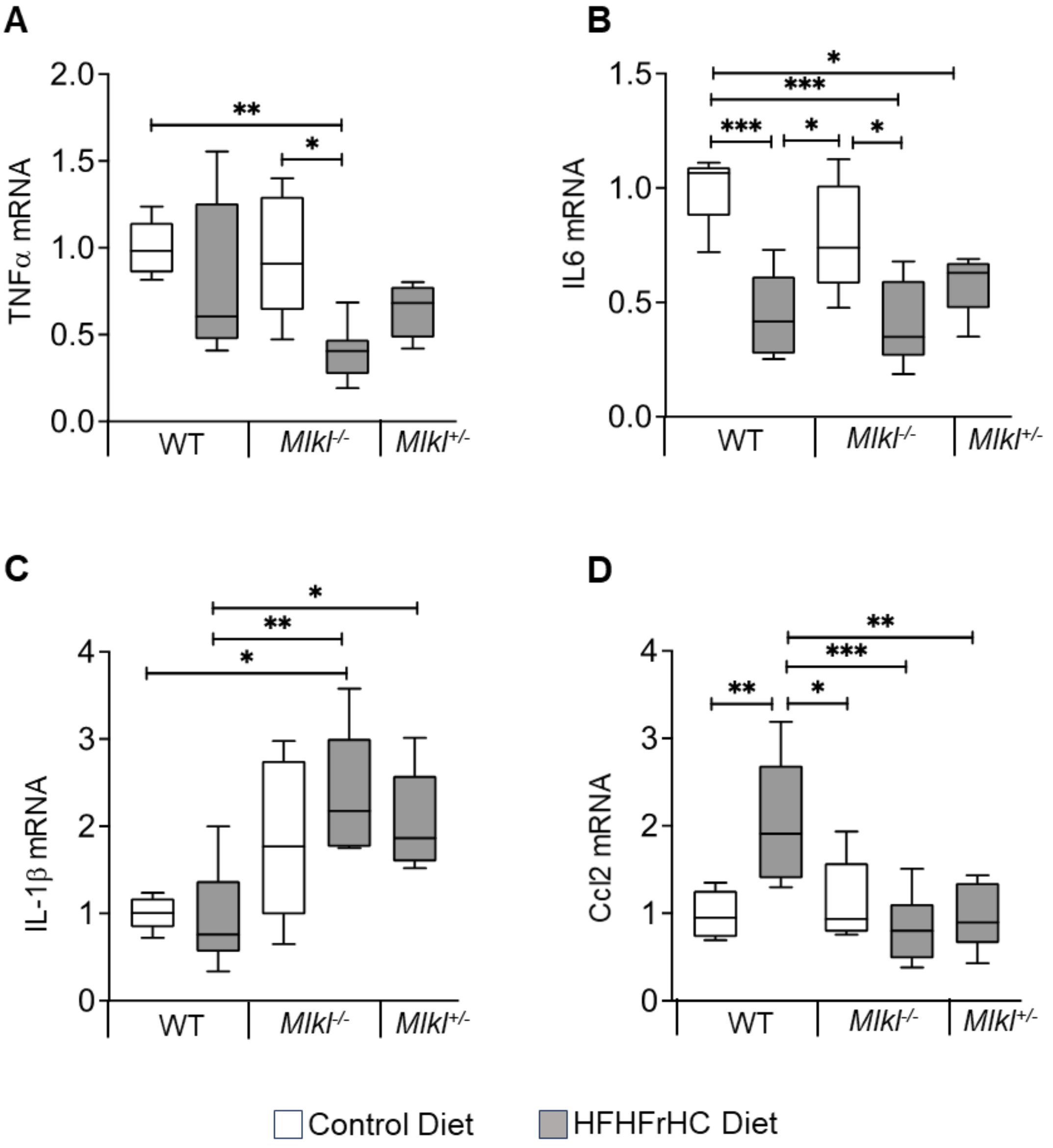

Figure S4: Effect of absence or reduction of ***Mlkl*** **on eWAT inflammatory cytokines.** Data from WT, *Mlkl*$^{-/-}$ or *Mlkl*$^{+/-}$ mice fed a CD (white box plots) or HFHFrHC diet (box plots): Transcript levels of inflammatory cytokines TNFα **(A)**, IL6 **(B)**, IL1β **(C)**, and Ccl2 **(D)**, normalized to β-microglobulin and represented as fold change relative to CD fed WT mice (n= 7-8 for WT; 5-8 for *Mlkl*$^{-/-}$ or *Mlkl*$^{+/-}$). Error bars are represented as mean± SEM. One-way ANOVA P0.05, * $p < 0.05$, ** $p < 0.005$, ***$p < 0.0005$.

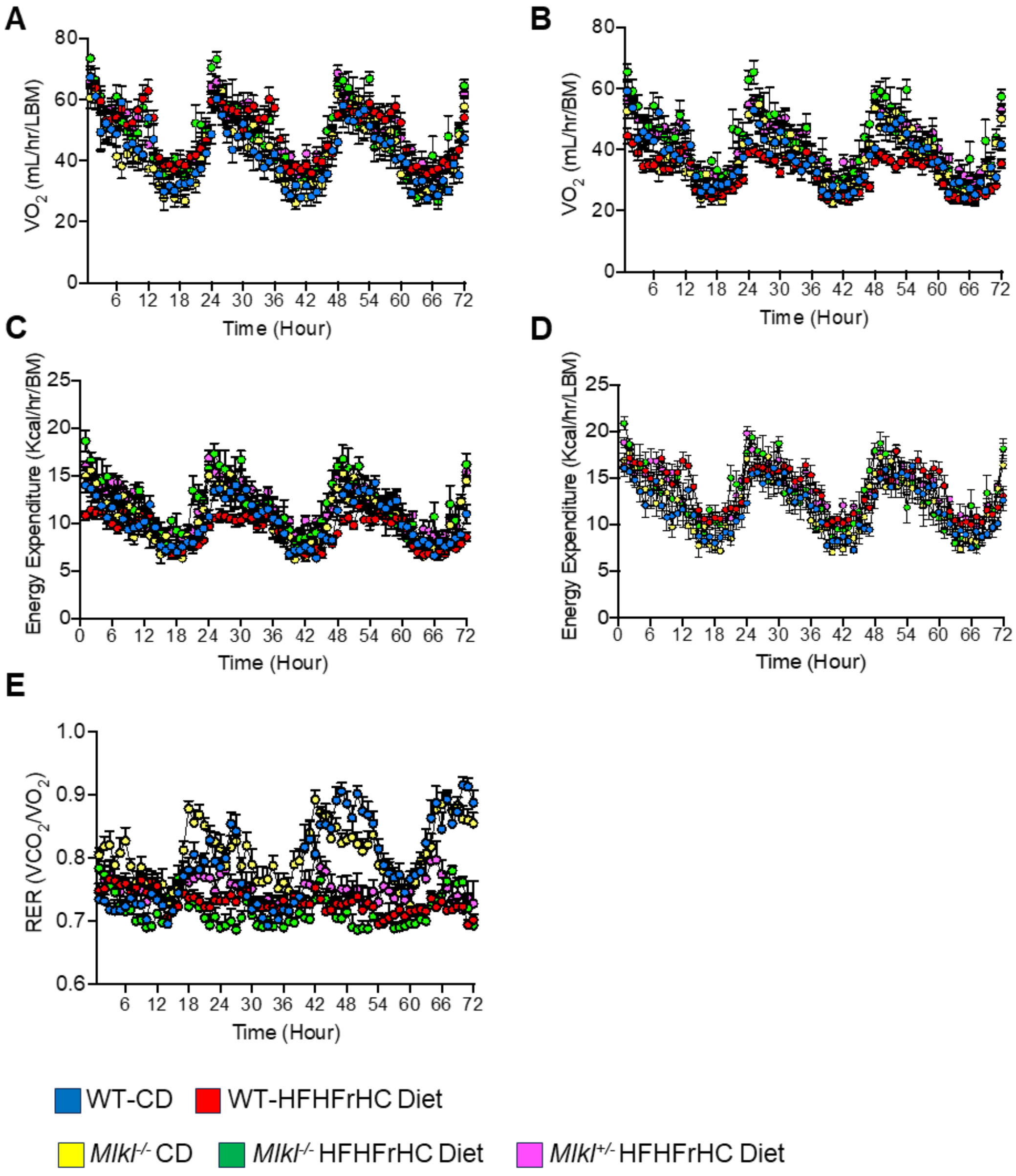

Figure S5: **Time course changes in oxygen consumption rate, energy expenditure, and respiratory quotient ratio of WT, *Mlkl*$^{-/-}$ or *Mlkl*$^{+/-}$ fed test diets.** Measures were recorded by indirect calorimetry over 72 hours in 6-hour intervals. Daily profiles of oxygen consumption rate normalized to total body mass (BM) **(A)** and lean body mass (LBM) **(B)**; Daily profiles of energy expenditure normalized to total body mass (BM) **(C)** and lean body mass (LBM) **(D)**; **(E)** Daily profile of respiratory quotient. In the figure legend- Blue: WT mice on LFD; Red: WT mice on HFD; Yellow: *Mlkl*$^{-/-}$ mice on LFD; Green: *Mlkl*$^{-/-}$ mice on HFD and Pink: *Mlkl*$^{+/-}$ mice on HFD. (n= 7-8 for WT; 5-8 for *Mlkl*$^{-/-}$ or *Mlkl*$^{+/-}$).
