# Supplementary material for: Non-Necroptotic Roles of MLKL in Diet-Induced Obesity, Liver Pathology, and Insulin Sensitivity: Insights from a High-Fat, High-Fructose, High-Cholesterol Diet Mouse Model
Source: Int J Mol Sci. 2024 Feb 28;25(5):2813. doi: 10.3390/ijms25052813 (PMC10931720; doi:10.3390/ijms25052813)
Supplement: Supplementary file 1 [file ijms-25-02813-s001.zip › Table S1.pdf]

Table S1 : Sequence of primers used in quantitative real time PCR analysis

Gene	Forward Sequence	Reverse Sequence
β-microglobulin	5′-CACTGACCGGCCTGTATGC-3′	5′-GGGTGGCGTGAGTATACTTGAAT-3′
Ccl2	5’-TTAAAAACCTGGATCGGAACCAA-3’	5’-GCATTAGCTTCAGATTTACGGGT-3’
Cebpα	5’-GCAAAGCCAAGAAGTCGGTGGA-3’	5’-CCTTCTGTTGCGTCTCCACGTT-3’
Cidea	5’-GGTGGACACAGAGGAGTTCTTTC-3’	5’-CGAAGGTGACTCTGGCTATTCC-3’
Cox8b	5’-GCGAAGTTCACAGTGGTTCC-3’	5’-GGAACCATGAAGCCAACGAC-3’
Hprt	5’-CTGGTGAAAAGGACCTCTCG-3’	5’-TGAAGTACTCATTATAGTCAAGGGCA-3’
IL-6	5’-TGGTACTCCAGAAGACCAGAGG-3’	5’-AACGATGATGCACTTGCAGA-3’
Il-1b	5’-AGGTCAAAGGTTTGGAAGCA-3’	5’-TGAAGCAGCTATGGCAACTG-3’
TNFα	5’-CACAGAAAGCATGATCCGCGACGT-3’	5’- CGGCAGAGAGGAGGTTGACTTTCT-3’
Pparα	5’-ACCACTACGGAGTTCACGCATG-3’	5′-GAATCTTGCAGCTCCGATCACAC-3’
Pparγ	5’-GTACTGTCGGTTTCAGAAGTGCC-3’	5′-ATCTCCGCCAACAGCTTCTCCT-3’
Prdm16	5′-AGCTGCGATTGAGGAAGAGCGA-3′	5′-CCTGGGTTCTGACGCCTTTGTT-3′
Ucp2	5′-CGTTCTGGGTACCATCCTAACC-3′	5′–CAAGCCTCCATCCAAGTGTCAC–3′
