# Supplementary material for: Non-Necroptotic Roles of MLKL in Diet-Induced Obesity, Liver Pathology, and Insulin Sensitivity: Insights from a High-Fat, High-Fructose, High-Cholesterol Diet Mouse Model
Source: Int J Mol Sci. 2024 Feb 28;25(5):2813. doi: 10.3390/ijms25052813 (PMC10931720; doi:10.3390/ijms25052813)
Supplement: Supplementary file 1 [file ijms-25-02813-s001.zip › Table S2.pdf]

Table S2: Expression of genes in the RT2 Profiler™ PCR Array for Mouse Cytokines & Chemokines

Genes	WT-CD		WT-HFHFrHC		$Mlkl^{-/-}$CD		$Mlkl^{-/-}$HFHFrHC		$Mlkl^{+/-}$HFHFrHC		Significant differences between the groups				
	Fold Change	SEM	Fold Change	SEM	Fold Change	SEM	Fold Change	SEM	Fold Change	SEM	WT-CD vs. WT-HFHFrHC	WT-CD vs. $Mlkl^{-/-}$ LFD	WT-HFHFrHC vs. $Mlkl^{-/-}$ HFHFrHC	WT-HFHFrHC vs. $Mlkl^{+/-}$ HFHFrHC	$Mlkl^{-/-}$ HFHFrHC vs. $MLKL^{+/-}$ HFHFrHC
Adipoq	1	0.18	0.14	0.03	0.38	0.15	1.17	0.26	1.43	0.33	No	No	Yes	Yes	No
Bmp2	1	0.28	0.71	0.08	1.18	0.16	1.29	0.36	0.94	0.16	No	No	No	No	No
Bmp4	1	0.23	1.51	0.24	2.71	0.07	6.94	0.29	3.94	0.69	No	No	Yes	Yes	Yes
Bmp6	1	0.19	0.51	0.10	1.52	0.16	1.77	0.41	1.26	0.24	No	No	Yes	No	No
Bmp7	1	0.24	1.81	0.38	1.90	0.27	2.52	0.22	3.77	0.33	No	No	No	Yes	No
Ccl1	1	0.24	1.99	0.90	0.87	0.19	10.93	2.99	4.46	2.61	No	No	Yes	No	No
Ccl11	1	0.20	2.79	0.49	1.67	0.13	32.96	2.37	5.56	1.25	No	No	Yes	No	Yes
Ccl12	1	0.24	4.87	0.89	1.02	0.12	13.00	5.31	3.83	2.23	No	No	No	No	No
Ccl17	1	0.27	4.10	1.64	0.87	0.20	1.61	0.67	1.04	0.49	No	No	No	No	No
Ccl19	1	0.12	2.03	0.26	1.01	0.07	5.08	0.38	4.94	2.00	No	No	No	No	No
Ccl2	1	0.41	6.25	1.85	2.89	1.67	15.26	4.84	3.48	1.49	No	No	No	No	Yes
Ccl20	1	0.26	2.57	1.12	0.68	0.10	14.13	2.92	1.78	1.04	No	No	Yes	No	No
Ccl22	1	0.33	42.07	0.47	3.71	0.76	46.30	8.68	96.27	18.10	Yes	No	No	Yes	Yes
Ccl24	1	0.27	1.74	0.85	1.22	0.08	5.65	1.21	4.63	1.98	No	No	No	No	No
Ccl3	1	0.26	5.52	2.77	1.06	0.25	13.87	3.34	11.91	3.48	No	No	No	No	No
Ccl4	1	0.21	4.81	1.95	1.05	0.16	12.87	2.22	12.45	2.97	No	No	No	No	No
Ccl5	1	0.10	2.60	0.29	1.30	0.41	6.64	0.82	5.82	2.60	No	No	No	No	No
Ccl7	1	0.57	6.99	2.94	5.14	0.05	4.74	3.51	0.89	0.13	No	No	No	No	No
Cd40lg	1	0.12	0.31	0.11	0.70	0.14	2.66	0.51	1.97	0.10	No	No	Yes	Yes	No
Cd70	1	0.24	0.70	0.09	0.87	0.19	1.63	0.40	1.75	0.21	No	No	No	Yes	No
Cntf	1	0.19	0.58	0.12	0.85	0.17	1.23	0.08	1.33	0.21	No	No	No	Yes	No
Csf1	1	0.16	0.89	0.14	0.84	0.19	2.27	0.35	2.05	0.70	No	No	No	No	No
Csf2	1	0.24	0.28	0.06	0.32	0.11	4.53	0.76	4.65	0.39	No	No	Yes	Yes	No
Csf3	1	0.09	0.98	0.04	2.40	0.43	1.68	0.42	13.45	2.89	No	No	No	Yes	Yes
Ctf1	1	0.18	0.92	0.21	1.42	0.22	1.95	0.37	1.99	0.53	No	No	No	No	No
Cx3cl1	1	0.18	2.58	1.27	1.95	0.25	13.15	3.80	4.99	1.72	No	No	Yes	No	Yes
Cxcl1	1	0.64	0.86	0.14	0.58	0.26	1.38	0.46	9.25	3.49	No	No	No	Yes	Yes
Cxcl10	1	0.73	1.60	0.17	1.28	0.26	2.77	0.47	1.93	0.64	No	No	No	No	No

Cxcl11	1	0.20	0.34	0.08	0.79	0.19	1.02	0.23	0.85	0.42	No	No	No	No	No
Cxcl12	1	0.22	0.96	0.06	1.46	0.22	2.17	0.22	1.98	0.03	No	No	Yes	Yes	No
Cxcl13	1	0.20	0.19	0.05	1.37	0.19	1.01	0.54	1.04	0.33	No	No	No	No	No
Cxcl16	1	0.16	2.14	0.54	1.23	0.17	7.14	1.72	1.60	0.47	No	No	Yes	No	Yes
Cxcl3	1	0.24	0.76	0.03	0.81	0.48	2.70	1.11	0.75	0.28	No	No	No	No	No
Cxcl5	1	0.24	0.76	0.03	1.04	0.22	17.12	6.24	1.75	0.21	No	No	Yes	No	Yes
Cxcl9	1	0.07	3.91	0.66	1.57	0.29	9.35	1.84	7.86	0.85	No	No	Yes	No	No
Fasl	1	0.25	1.44	0.43	0.85	0.18	4.90	1.89	0.41	0.05	No	No	No	No	Yes
Gpi1	1	0.16	0.50	0.09	0.73	0.13	1.38	0.33	1.01	0.28	No	No	No	No	No
Hc	1	0.32	0.91	0.19	1.69	0.20	2.23	0.54	2.01	0.24	No	No	Yes	No	No
Ifna2	1	0.40	12.65	3.70	1.34	0.12	4.58	0.67	15.04	1.96	Yes	No	No	No	Yes
Ifng	1	0.53	2.43	0.25	1.24	0.12	10.96	2.06	0.76	0.11	No	No	Yes	No	Yes
Il10	1	0.24	4.18	1.03	1.16	0.17	5.06	0.66	11.32	3.37	No	No	No	Yes	No
Il11	1	0.24	5.28	0.89	3.98	0.94	3.80	0.67	1.75	0.21	Yes	Yes	No	Yes	No
Il12a	1	0.24	1.69	0.09	2.16	0.05	3.64	0.27	1.75	0.21	No	Yes	Yes	No	Yes
Il12b	1	0.24	15.35	3.98	2.65	0.37	35.37	3.71	20.69	4.08	Yes	No	Yes	No	Yes
Il13	1	0.24	1.43	0.56	5.87	1.13	7.08	1.66	1.75	0.21	No	Yes	Yes	No	Yes
Il15	1	0.33	1.43	0.40	1.18	0.11	4.77	0.71	2.93	0.56	No	No	Yes	No	No
Il16	1	0.29	1.04	0.16	1.10	0.27	3.15	0.45	2.61	0.61	No	No	Yes	No	No
Il17a	1	0.24	0.76	0.03	1.02	0.12	3.55	0.54	1.75	0.21	No	No	Yes	Yes	Yes
Il17f	1	0.14	0.86	0.09	1.56	0.11	2.82	1.01	6.54	3.60	No	No	No	No	No
Il18	1	0.32	0.65	0.19	1.76	0.30	1.85	0.39	1.51	0.40	No	No	No	No	No
Il1a	1	0.21	1.00	0.20	1.38	0.35	2.94	0.77	3.11	1.32	No	No	No	No	No
Il1b	1	0.22	0.93	0.26	1.48	0.03	4.68	1.01	1.81	0.70	No	No	Yes	No	Yes
Il1rn	1	0.31	16.20	3.10	1.74	0.38	21.29	3.26	9.40	1.57	Yes	No	No	No	Yes
Il2	1	0.24	1.36	0.45	0.87	0.19	7.58	3.98	1.75	0.21	No	No	Yes	No	Yes
Il21	1	0.24	0.76	0.03	0.87	0.19	3.47	0.87	1.75	0.21	No	No	Yes	No	Yes
Il22	1	0.24	0.76	0.03	1.01	0.10	1.11	0.15	1.75	0.21	No	No	No	Yes	No
Il23a	1	0.28	0.67	0.17	0.52	0.16	1.35	0.46	1.36	0.29	No	No	No	No	No
Il24	1	0.24	0.76	0.03	0.87	0.19	0.54	0.29	1.75	0.21	No	No	No	Yes	Yes
Il27	1	0.12	2.79	0.33	1.44	0.14	4.89	0.18	5.41	0.89	Yes	No	Yes	Yes	No
Il3	1	0.24	0.76	0.03	1.26	0.30	2.36	0.06	1.75	0.21	No	No	Yes	Yes	No
Il4	1	0.28	1.73	0.35	2.78	1.02	4.12	1.48	2.43	1.89	No	No	No	No	No

Il5	1	0.24	4.49	1.17	2.28	0.10	3.01	0.58	1.75	0.21	Yes	No	No	No	No
Il6	1	0.24	2.29	0.34	2.48	0.31	5.70	1.05	1.75	0.21	No	No	Yes	No	Yes
Il7	1	0.38	1.39	0.27	0.60	0.01	6.29	0.71	2.98	0.70	No	No	Yes	No	Yes
Il9	1	0.24	2.32	0.52	0.49	0.24	1.11	0.13	1.75	0.21	Yes	No	No	No	No
Lif	1	0.24	5.47	1.86	4.12	0.17	35.60	4.41	1.75	0.21	No	No	Yes	No	Yes
Lta	1	0.24	1.97	0.60	0.87	0.19	11.68	4.69	10.18	4.51	No	No	No	No	No
Ltb	1	0.33	2.62	0.90	1.36	0.39	10.61	3.03	5.18	0.83	No	No	Yes	No	Yes
Mif	1	0.11	1.11	0.18	1.41	0.27	1.47	0.19	1.12	0.10	No	No	No	No	No
Mstn	1	0.24	0.76	0.03	0.53	0.22	3.69	2.11	1.75	0.21	No	No	No	No	No
Nodal	1	0.16	0.49	0.11	0.43	0.06	2.23	0.53	2.49	1.09	No	No	No	No	No
Osm	1	0.24	4.90	1.01	1.81	0.33	16.98	4.20	1.75	0.21	No	No	Yes	No	Yes
Pf4	1	0.21	2.11	0.81	0.84	0.13	5.34	0.99	1.87	0.87	No	No	Yes	No	Yes
Ppbp	1	0.28	1.41	0.31	0.91	0.19	2.57	0.72	3.65	1.30	No	No	No	No	No
Spp1	1	0.09	6.09	2.01	1.71	0.45	53.54	6.57	2.94	1.58	No	No	Yes	No	Yes
Tgfb2	1	0.18	1.46	0.44	0.50	0.04	3.56	0.84	1.33	0.37	No	No	Yes	No	Yes
Thpo	1	0.06	0.57	0.04	1.08	0.09	1.58	0.08	1.22	0.48	No	No	No	No	No
Tnf	1	0.52	3.30	0.82	1.29	0.52	11.28	4.10	6.64	2.18	No	No	Yes	No	No
Tnfrsf11b	1	0.19	1.90	0.24	1.18	0.26	2.43	0.87	1.76	0.36	No	No	No	No	No
Tnfsf10	1	0.55	1.04	0.44	1.46	0.49	3.20	0.36	2.23	0.69	No	No	Yes	No	No
Tnfsf11	1	0.26	0.76	0.03	0.73	0.14	0.98	0.06	1.51	0.18	No	No	No	Yes	No
Tnfsf13b	1	0.17	2.40	0.27	1.07	0.04	10.11	1.54	4.61	0.25	No	No	Yes	No	Yes
Vegfa	1	0.31	0.50	0.07	0.84	0.19	0.67	0.09	1.04	0.36	No	No	No	No	No
Xcl1	1	0.24	8.08	0.64	3.67	0.58	16.19	3.12	36.19	7.80	No	No	No	Yes	Yes
