# Supplementary material for: Non-Necroptotic Roles of MLKL in Diet-Induced Obesity, Liver Pathology, and Insulin Sensitivity: Insights from a High-Fat, High-Fructose, High-Cholesterol Diet Mouse Model
Source: Int J Mol Sci. 2024 Feb 28;25(5):2813. doi: 10.3390/ijms25052813 (PMC10931720; doi:10.3390/ijms25052813)
Supplement: Supplementary file 1 [file ijms-25-02813-s001.zip › Table S3.pdf]

Table S3: Composition of diet used in the study

Product	Control Diet (g)	Control Diet (kcal %)	HFHFrHC Diet (g)	HFHFrHC Diet (kcal %)
**Protein**	19	20	22	20
**Carbohydrate**	67	70	45	40
**Fat**	4	10	20	40
**Total**		100		100
**kcal/gram**		3.8		4.5
**Composition**	**Control Diet (g)**	**Control Diet (kcal)**	**HFHFrHC Diet (g)**	**HFHFrHC Diet (kcal %)**
**Protein**				
Casein	200	800	200	800
L-Cystine	3	12	3	12
**Carbohydrate**				
Corn Starch	350	1400	0	0
Maltodextrin 10	85	340	100	400
Fructose	0	0	200	800
Dextrose	169	676	0	0
Sucrose	96	384	96	384
Cellulose	50	0	50	0
**Fat**				
Soybean Oil	25	225	25	225
Primex Shortening	0	0	0	0
Palm Oil	0	0	135	1215
Lard	20	180	20	180
Mineral Mix S10026	10	0	10	0
DiCalcium Phosphate	13	0	13	0
Calcium Carbonate	5.5	0	5.5	0
Potassium Citrate, 1H2O	16.5	0	16.5	0
Vitamin Mix V10001	10	40	10	40
Choline Bitartrate	2	0	2	0
**Cholesterol**	0	0	18	0
